# Supplementary material for: Effects of global and domestic tobacco control policies on cigarette consumption per capita: an evaluation using monthly data in China
Source: BMJ Open. 2019 Jun 19;9(6):e025092. doi: 10.1136/bmjopen-2018-025092 (PMC6588959; doi:10.1136/bmjopen-2018-025092)
Supplement: Author's manuscript [file bmjopen-2018-025092.draft_revisions.pdf]

BMJ Open is committed to open peer review. As part of this commitment we make the peer review history of every article we publish publicly available.

When an article is published we post the peer reviewers' comments and the authors' responses online. We also post the versions of the paper that were used during peer review. These are the versions that the peer review comments apply to.

The versions of the paper that follow are the versions that were submitted during the peer review process. They are not the versions of record or the final published versions. They should not be cited or distributed as the published version of this manuscript.

BMJ Open is an open access journal and the full, final, typeset and author-corrected version of record of the manuscript is available on our site with no access controls, subscription charges or pay-per-view fees (http://bmjopen.bmj.com).

If you have any questions on BMJ Open's open peer review process please email info.bmjopen@bmj.com

BMJ Open

Global and Domestic Tobacco Control Policies Combined Have Halted the Growing Trend of Cigarette Consumption in China: Findings from 17 Years of Monthly Data

Journal:	*BMJ Open*
Manuscript ID	bmjopen-2018-025092
Article Type:	Research
Date Submitted by the Author:	08-Jul-2018
Complete List of Authors:	Xu, Xiaoxin; Beijing Normal University, School of Social Development and Public Policy<br>Zhang, Xiulan; Beijing Normal University, School of Social Development and Public Policy<br>Hu, Teh-wei; Public Health Institute, Center for International Tobacco Control<br>Miller, Leonard; University of California, School of Social Welfare<br>XU, Mengnan; Beijing Normal University, School of Social Development and Public Policy
Keywords:	global health, smoking, global and domestic health policies

SCHOLARONE™
Manuscripts

For peer review only

Global and Domestic Tobacco Control Policies Combined Have Halted the Growing Trend of Cigarette Consumption in China: Findings from 17 Years of Monthly Data Word Count: 3338

Xu Xiaoxin, Xiulan Zhang, Teh-wei Hu, Leonard Miller, Mengnan Xu

Authors

Xiaoxin Xu, Ph.D.,

School of Social Development and Public Policy

Beijing Normal University, China

Email: xuxiaoxin@bnu.edu.cn

Xiulan Zhang, Ph.D.,

School of Social Development and Public Policy

Beijing Normal University, China

Email: zhang99@bnu.edu.cn

Teh-wei Hu, Ph.D.,

Center for International Tobacco Control,

Public Health Institute, Oakland, CA, USA

Email: thu@berkeley.edu

Leonard S. Miller, Ph.D.,

School of Social Welfare

University of California, Berkeley, CA, USA

Email: lsmiller2331@gmail.com

Mengnan Xu, B.S.,

For peer review only

For peer review only - http://bmjopen.bmj.com/site/about/guidelines.xhtml

School of Social Development and Public Policy,

Beijing Normal University, China

Email: pumpkinggg@126.com

Correspondence: Xiulan Zhang, School of Social Development and Public Policy, Beijing Normal University, Beijing, China, 19 Xinjiekouwai Street, Haidian District, Beijing, 100875, China. Tel: (86-10) 5880-1512; Fax: (86-10) 5880-0366; Email: zhang99@bnu.edu.cn

For peer review only

For peer review only - http://bmjopen.bmj.com/site/about/guidelines.xhtml

Abstract

Introduction China consumes 44% of the world's cigarettes. Robust tobacco control policies are needed to stop the continuing trend of increased cigarette consumption.

Methods Monthly data on cigarette consumption per capita from January 2000 to June 2017, a period of 17·5 years or 210 months, are used to estimate the impact of specific policies on China's tobacco consumption. The policies studied include the World Health Organization's (WHO) Framework Convention on Tobacco Control (FCTC), national tobacco-related policy changes, and two tobacco tax increases implemented in China during the study period. Segmented regression analysis is used to estimate the immediate effect of the policies studied and changes in the time trends resulting from these policy changes.

Findings The impact of national policy changes in China is almost 20 times greater than the impact of the WHO's FCTC treaty itself, and national policy changes in tobacco control are a determining factor in reversing the trend of increased tobacco consumption in China. The 2015 tax increase, which raised retail cigarette prices, produced both immediate and trend effects, with a total marginal effect 7·8 times that of the 2009 tax increase, which did not result in higher cigarette prices for the consumer.

Interpretations Translating global social norms of tobacco control into national policies will generate much higher impact on average cigarette consumption, and tobacco taxes that are reflected in the retail prices will be more effective in reducing the consumption of cigarettes.

Strengths and limitations of this study

1. To the best of our knowledge, this study is the first systematic evaluation of the impact of

For peer review only - http://bmjopen.bmj.com/site/about/guidelines.xhtml

both domestic and global tobacco control policies on tobacco consumption in China.

2. The study compares the effectiveness of the global FCTC and domestic policies to reduce cigarette consumption in China over a period 17·5 years.

3. The data used for the policy evaluation covers the periods from no tobacco control policies in China to the implementation of FCTC policies to the changed national policies to the specific tax increases enacted in China in 2009 and 2015.

4. Using the interrupted time series model, the study not only examines the immediate impact of each policy on tobacco consumption, but also the policy impact on tobacco consumption trends.

For peer review only

For peer review only - http://bmjopen.bmj.com/site/about/guidelines.xhtml

Introduction

The 315 million smokers in China consume 44% of the world's cigarettes, and their average consumption is 2·3 times the world average.[1] Tobacco use increases medical expenses by billions.[1] Each year, one million people in China, many of them young, die of tobacco-related diseases.[2] China's rapid economic development in the past 40 years has been accompanied by significant growth in the country's total cigarette consumption. In 2000, the China National Tobacco Corporation (CNTC), the state-owned tobacco monopoly, sold 76·92 billion packs of cigarettes;[3] by 2014, the number had grown to 127·48 billion packs,[4] an increase of 65·8 %.

China signed the World Health Organization's (WHO) Framework Convention on Tobacco Control (FCTC) in 2003; the China National People's Congress (CNPC) ratified the treaty in 2005, and China began implementing the FCTC in 2006. In this paper, however, we refer to the WHO framework as an international policy. While the Chinese government has made some effort to control tobacco use, strong interference from the China State Tobacco Monopoly Administration (STMA), which owns CNTC, in favoring economic concerns over social concerns has led to slow development and implementation of the full tobacco control policy measures. [5-10]

Between 2006 and 2015, China increased tobacco taxes twice. The first tax increase was introduced in May 2009 and was not reflected in cigarette retail prices directly. As a result, the increase had minimal immediate impact on consumers, but it might have more long term impact by changing cigarette product structure and consequent average price raising.[11] The 2009 adjustment raised the ad valorem tax from 45% to

For peer review only - http://bmjopen.bmj.com/site/about/guidelines.xhtml

56% at the producer price level for class A cigarettes and from 30% to 36% for class B cigarettes. The new policy also introduced a new 5% ad valorem tax at the wholesale price level.[12,13]The intent of this 2009 adjustment was to raise government revenue from CNTC, China's tobacco producer, not to serve as a tobacco control policy instrument. Under the new scheme, the government forbade changes in the retail prices of cigarettes.[13-15] The policy was introduced primarily to counteract the impact of the global financial crisis on government revenue. Before this tax increase in May 2009, China's public revenue had declined for the previous seven months. Between January and April 2009, public revenue decreased 9·9% while public spending increased 31·7%. The financial pressure prompted the government to raise the tobacco tax. In other words, this policy was driven by an economic goal, and because the policy forbade the tobacco industry from adding the tax increase to the retail price of cigarettes, the social goal was not considered at all.[13,14]

The second tobacco tax increase occurred in May 2015. Unlike the 2009 tax adjustment, the 2015 adjustment moved the increase from the tax base at the wholesale price level to the retail price level, a significant step away from the 2009 increase and toward China's tobacco control agenda.[16] The 2015 tax increase initiated a 0·10 RMB tax per pack at the wholesale price level and increased the ad valorem tax from 5% to 11%, a 6% increase also at the wholesale price level. However, this time, the Chinese government allowed the tobacco industry to shift this new tax increase to the retail price, an estimated 10% increase in the retail price of cigarettes.[17]

For peer review only - http://bmjopen.bmj.com/site/about/guidelines.xhtml

China's new administration came into power in 2013. Support from its top leader, President Xi, began to change the policy direction of tobacco control in China.[18] The national policy change began with the anti-corruption campaign, which was aimed at the problem of corruption within the party, state, and business sectors.[19] In November 2013, the government forbade the purchase of cigarettes using public revenue. A month later additional policies were announced that prohibited cadres from smoking in public places. This significant policy initiative can be considered a major government effort to change the social norm of smoking habits in China.

Insert Figure 1 Here

One factor that has influenced the trend of increased cigarette consumption in China is the rapid growth of personal income. China has experienced the largest economic transformation in human history. Following the 1978 economic reform, the Chinese economy grew around 9·5% each year, becoming the second largest economy in the world. In recent years, China's economic performance has remained at a relatively high level of 7% growth. While the income of people in China also has increased significantly, an increase in cigarette prices has not accompanied the GDP growth, thus making cigarettes more affordable over time.[20,21]

Waiting for China to take robust measures to control its tobacco use, change the social norm and policy landscape, reduce the institutional barriers created by the China

For peer review only - http://bmjopen.bmj.com/site/about/guidelines.xhtml

STMA, and counteract the increased consumption of cigarettes resulting from income growth is a long and frustrating process.

The purpose of this paper is to estimate the relative impacts of four tobacco control policy interventions on tobacco consumption in China: the international WHO policy (FCTC), the government's 2013 national policy forbidding use of general revenue to purchase cigarettes and smoking in public by cadres, and the tax increases of 2009 and 2015. This is the first study to estimate the combined impact of international and domestic tobacco control policy changes on long-term trends in tobacco consumption in China.

Methods

Data

We used the monthly data on cigarette sales from January 2000 to June 2017, a total of 210 months of data reported by CNTC. Sales data, collected by CNTC, are based on the purchases of retailers, so the exact monthly sales are determined by the dates when retailers buy from cigarette distributors.

Patient and Public Involvement

There is no patient and public involvement of the data collection.

Between 2000 and 2016, China's total population increased by 9·1%. To adjust for the effect of population growth on cigarette sales, this study uses the average packs of cigarettes consumed each month per capita.[22] To estimate policy impacts, we include the GDP growth rate, the timing of policy interventions, and trends initiated by each of the four policy interventions studied here.

For peer review only - http://bmjopen.bmj.com/site/about/guidelines.xhtml

During the study period, several tobacco control policies were implemented in China. As discussed in the introduction section, the first was the ratification of WHO's FCTC, the implementation of which began in January 2006. In May 2009, China raised cigarette taxes, but the increase was not reflected in the retail price. In 2013, tobacco control received top leadership support. In November, a national policy was issued forbidding government funds from being used to purchase cigarettes for officials, and a month later, in December, the Central Committee of the Communist Party of China (CCCPC) and the State Council jointly issued a policy prohibiting cadres from smoking in public places. This national policy targeted party officials and government agencies. In May 2015, China again raised cigarette taxes, this time allowing retail prices to rise.

Since the implementation of WHO's FCTC, smoke-free policies have been established in different cities or regions of China. The Beijing Municipal Government passed the strictest smoke-free regulation in May 2015. But a national smoke-free law has not passed. Therefore, the effect of smoke-free policies is an unmeasured effect in the model.

We divided the analysis into five time periods: (1) before the FCTC was implemented (January 2000 to December 2005); (2) between implementation of the FCTC and the first tax policy adjustment (January 2006 to April 2009); (3) between the first tax policy adjustment and implementation of the national policy change (May 2009 to October 2013); (4) between implementation of the national policy change and the

For peer review only - http://bmjopen.bmj.com/site/about/guidelines.xhtml

second tax policy adjustment (November 2013 to April 2015); and (5) the period after all policies were implemented (May 2015 to June 2017).

Table 1 presents descriptive statistics of the GDP growth rates and average packs of cigarettes consumed during each of the five periods studied.

Table 1 Average cigarette consumption and GDP growth in different periods

Period	Policies	Number of Months	Average Consumption (Pack/month)	Average GDP Growth (%)
Jan 2000 to Dec 2005	No Policies	72	5·66 (0·57)	9·54 (1·06)
Jan 2006 to Apr 2009	FCTC Only	40	6·78 (1·22)	11·86 (2·02)
May 2009 to Oct 2013	FCTC/Tax1	54	7·43 (1·83)	8·87 (1·11)
Nov 2013 to Apr 2015	FCTC/Tax1/National	18	7·69 (2·14)	7·32 (0·25)
May 2015 to Jun 2017	All Policies	26	7·20 (1·56)	6·81 (0·10)

The GDP growth rates during the 17 years for which we have data reached 14·16% in 2007 and then dropped to 6·7% in 2016. The average growth rate over the analysis period was 9·36%. The decline in GDP growth rates began in 2012.

Statistical Analysis

Segmented regression analysis of interrupted time series is an effective statistical method to evaluate longitudinal effects of time-delimited interventions,[23] and it is widely used in assessing policy impact. In this model, two parameters are estimated for each intervention studied: level and trend. The level parameter defines the y-intercept, which is the immediate effect of the intervention on the outcome. The time trend interaction with the intervention variable is the rate of change (the slope), which measures the gradual change of the outcomes due to the intervention.[24,25]

For peer review only - http://bmjopen.bmj.com/site/about/guidelines.xhtml

For peer review only

We estimated the segmented regression model using SAS AUTOREG procedures to assess the longitudinal impact of tobacco control policies on the average cigarette consumption per month per capita. We estimated levels and trends of the four interventions: FCTC (2005), first taxation (2009), national policies (2013), and second taxation (2015). The monthly pattern of sales was adjusted by the AR parameters in AUTOREG procedure.

Results

Table 2 presents an estimation of the model describing average sales of packs of cigarettes consumed per person per month. Overall, the model is very significant with a total R-squared of 0·9416. The transformed R-squared is 0·995, indicating an extremely high fit of the model and the existence of autocorrelation.

Table 2: Auto-regression model estimate of the per-capita monthly cigarette consumption

Maximum-Likelihood Estimates of the Model			
SSE	30·94	DFE	187
MSE	0·165	Root MSE	0·407
SBC	338·86	AIC	261·88
MAE	0·27	AICC	267·81
MAPE	4·04	HQC	293·00
Log Likelihood	-107·94	Observations	210
Total R^2			0·9416
Transformed R^2			0·9950
**Parameter Estimates**			
Variable	Estimate	t Value	Pr > |t|

For peer review only - http://bmjopen.bmj.com/site/about/guidelines.xhtml

Intercept	4·8353	70·45	<·0001
GDP Growth	0·0180	2·10	0·0371
Time	0·0184	32·99	<·0001
FCTC	-0·1101	-2·53	0·0121
FCTC_time	0·0046	2·37	0·0189
Tax1	-0·0462	-1·38	0·1686
Tax1_time	-0·0097	-7·55	<·0001
National	-0·3056	-5·63	<·0001
National_time	0·0078	1·72	0·0871
Tax2	-0·4309	-6·39	<·0001
Tax2_time	-0·0382	-8·30	<·0001
AR1	0·6277	8·87	<·0001
AR2	0·5907	8·04	<·0001
AR3	0·5834	7·83	<·0001
AR4	0·5588	7·33	<·0001
AR5	0·5435	7·00	<·0001
AR6	0·5458	6·85	<·0001
AR7	0·5412	6·72	<·0001
AR8	0·5397	6·72	<·0001
AR9	0·5546	6·98	<·0001
AR10	0·5662	7·23	<·0001
AR11	0·5644	7·32	<·0001
AR12	-0·3572	-4·83	<·0001

The time effect is positive and significant, indicating the urgency to interrupt the trend of increasing cigarette consumption in China to reduce the burden of diseases and death due to smoking.

For peer review only

For peer review only - http://bmjopen.bmj.com/site/about/guidelines.xhtml

The parameter estimate shows that GDP growth has a positive and statistically significant impact on monthly cigarette consumption. GDP growth indicates a macro income effect; this effect conforms with the literature that the income elasticity of cigarette consumption is positive.

In terms of the impact of tobacco control policies, implementation of the FCTC in 2006 resulted in an immediate reduction in the number of cigarettes consumed. However, after the initial reduction, consumption again rose over time, indicating that either the tobacco industry developed new strategies to counteract the FCTC policy or consumers resumed the intensity of their smoking habits after the initial reaction to the macro-policy change.

Similar to what happened after implementation of the international treaty, when the CCCPC and the State Council jointly issued a national policy on cadres and governments in 2013, consumption of cigarettes dropped immediately. The drop following announcement of the national policy was about three times the drop in consumption after implementation of the FCTC. Again, similar to what happened after implementation of the FCTC, the trend after the change in national policy was positive subsequent to the drop, but not statistically significant. This finding shows that while the 2013 national policy changes aimed at changing cigarette-related social norms could immediately impact average sales, the after effect was counteracted by either consumer habits or more aggressive marketing strategies by the tobacco industry.

For peer review only - http://bmjopen.bmj.com/site/about/guidelines.xhtml

As for China's two tobacco tax initiatives, the coefficient of the Tax 1 (2009) variable is not statistically significant, as one would have expected. Over time, the tobacco industry restructured its market share but the magnitude of the coefficient of the time and tax interaction term is still very small, though statistically significant. However, the coefficient of the Tax 2 (2015) variable and its time trend interaction term are both statistically significant with a magnitude four times larger than the FCTC effect and 50 percent higher than the national policy effect. As shown from the coefficient and its interaction term, the 2015 tobacco tax increase (Tax 2) essentially reduced per capita monthly consumption by 0·43 pack initially and then continued to reduce consumption by 0·04 pack per capita per month over time.

The implementation of both tax increases (2009 and 2015) resulted in similar initial effects and time trends. The initial effect of the second (2015) tax increase, aimed at wholesale and retail prices, was about ten times the initial effect of the 2009 tax increase. The trend effect of the 2015 policy was about four times the trend effect of the first tax increase. In addition, unmeasured smoke-free model effects might have contributed to the big impact of the 2015 tax increase.

This finding indicates that unless specific policies are targeted at smokers, generalized policies advocating tobacco control may result in some immediate effects, but they won't be able to change smokers' behavior over time.

Tax increases are much more effective at changing smokers' behavior than generalized policies.   This was true of even the first tax increase (in 2009), which was not reflected in the retail price of cigarettes. When the tax increase was factored

For peer review only - http://bmjopen.bmj.com/site/about/guidelines.xhtml

into the retail price (in 2015), the impact on cigarette consumption was much larger and was sustained over time.

Figure 2 presents China's average monthly packs of cigarettes consumed per capita per year from 2000-2017 with and without accounting for tobacco control policies. Before the FCTC policy was implemented, between 2000 and 2005, average consumption increased from 5.1 packs to 6·3 packs per capita per month, an increase of 23·5% in six years.

Between 2006 and 2013, monthly cigarette consumption grew from 6·4 packs to 7·7 packs, an increase of 20·3% in seven years. Consumption then began to decrease in 2013. By the end of 2016, the average number of packs of cigarettes consumed monthly had dropped from 7·7 to 7·2, a 6·5% decrease in three years. Without the tax increase, average consumption was predicted at 8·6 packs, 16·3% higher than with the tax increase.

The 2013 policy announcements by the Chinese national government changed smoking-related social norms. Combined with the global FCTC intervention and the first tax increase (2009), the growing trend of cigarette consumption per month per capita in China finally began to decline, and the second tax increase (2015) had a much bigger impact on the downward trend.

Insert Table 2 Here

For peer review only - http://bmjopen.bmj.com/site/about/guidelines.xhtml

Based on the model estimates, we calculated the total impact of various policies on the average number of packs of cigarettes consumed per month. Table 3 presents the percentage change in average monthly consumption of cigarettes, with and without tobacco control policies. The percentage effect is calculated as follows:

$$\% \text{ Change} = (Y\hat{}_{policies=1} - Y\hat{}_{policies=0})/Y\hat{}_{policies=0}$$

Table 3: Impact of tobacco control policies on average monthly cigarette consumption per capita

Policy Period	Predicted Monthly Consumption in Packs of Cigarettes with Policies ($Y\hat{}_{policies=1}$)	Predicted Monthly Consumption in Packs of Cigarettes without Policies ($Y\hat{}_{policies=1}$)	% Change ($Y\hat{}_{policies=1}$ $-Y\hat{}_{policies=0}$) $/Y\hat{}_{policies=0}$	% Marginal Change
No Policies	5·680	5·680	0·00%	0·00%
FCTC Only	6·737	6·754	-0·25%	-0·25%
FCTC/Tax1	7·450	7·566	-1·54%	-1·29%
FCTC/Tax1/National	7·668	8·202	-6·51%	-4·97%
FCTC/Tax1/National/Tax2	7·176	8·598	-16·54%	-10·03%

Table 3 shows that during the 40-month period when only the FCTC policy was in effect (January 2006 through April 2009), average cigarette consumption dropped 0·25%, due mainly to the initial impact of the FCTC.

During the 54-month period that includes implementation of the FCTC and the first tax increase (May 2009 through October 2013), average consumption dropped 1·54%, and the marginal effect of the 2009 tax increase was -1·29%, indicating a very limited effect when the tax increase was not factored into the retail price.

During the 18-month period following issuance of the national policies (November 2013 through April 2015), but prior to the second tax increase instituted in May 2015,

For peer review only - http://bmjopen.bmj.com/site/about/guidelines.xhtml

the average consumption of cigarettes dropped 6·51%, and the national policy changes alone reduced monthly consumption by 4·97%.

After the second tax increase announced in May 2015, a big decline occurred in cigarette consumption. In the 26-month period following this tax increase (May 2015 through June 2017), the average consumption of cigarettes dropped 16·54%, due mainly to the effect of the second tax increase, which alone brought down average monthly consumption by 10·03%.

Table 4 presents the predicted effects of the four policies studied on cigarette consumption measured in million packs.

Table 4: Impact of tobacco control policies on cigarettes consumption in million packs

Period	FCTC	1st Taxation	National	2nd Taxation	All Policies
Jan 2006 to Apr 2009	893	0	0	0	893
May 2009 to Oct 2013	1,377	7,106	0	0	8,484
Nov 2013 to Apr 2015	505	2,606	10,042	0	13,153
May 2015 to Jun 2017	771	3,980	15,336	30,949	51,036
Total	3,547	13,693	25,377	30,949	73,567

Between January 2006 and June 2017, China consumed 1·348 trillion packs of cigarettes. The reduction in total consumption attributable to the policy changes was 73·6 billion packs, which is 5·18% of the sales predicted without policy interventions. Implementation of the FCTC decreased consumption by 3·5 billion packs, the first tax increase (2009) reduced consumption by 13·7 billion, the national policy announcements decreased it by 25·4 billion packs, and the largest reduction came

For peer review only - http://bmjopen.bmj.com/site/about/guidelines.xhtml

from the second tax increase (2015), including unmeasured local smoke-free policies--almost 31 billion packs in just 26 months.

From announcement of the 2013 national policy change through June 2017, China reduced the sales of cigarettes by 64·2 billion packs, a 12·57% reduction in the average consumption of cigarettes in China.

Discussion

The World Health Organization's FCTC, an international treaty, aims to provide a roadmap to address the global tobacco epidemic using effective measures and strategies. China ratified the treaty in November 2005 and began implementation in January 2006.

This study finds that the impact of national policy changes has been almost 20 times larger than the impact of the WHO's FCTC treaty itself, and that national tobacco control policy changes in China have been a determining factor in reversing the increasing trend of tobacco consumption. In other words, implementing an international treaty requires national policy and social norm changes to achieve the goal of reducing tobacco consumption. Ratification of the treaty alone without domestic policy implementation will have a very minimal effect.

The process of integrating global social norms with domestic policy change took exactly 10 years in China (November 2003 to November 2013).[26] Our study finds that after the immediate effects of the policy changes were noted, the powerful STMA developed countermeasures to dilute the impact of the policy changes. This finding

For peer review only - http://bmjopen.bmj.com/site/about/guidelines.xhtml

confirms the challenges faced by and the persistence required for the global and national tobacco control communities. [27,28]

Between 2006 and 2013, although the government raised the tobacco tax in May 2009, the economic goal of increasing government revenue overpowered the social goal of reducing tobacco consumption. The tax increase did not result in higher cigarette prices for the consumer, thus minimizing the impact of this policy on consumption.[14,29]

When the 2015 tax policy raised retail cigarette prices, both immediate and trend effects were very significant, and the total marginal effect was 7·8 times that of the 2009 tax increase. This finding indicates that tobacco control policies should be more robust and target consumers

more directly through higher prices and tougher smoke-free regulations. Because China has no national smoke-free law, and the impact of various local smoke-free regulations on national cigarette consumption is difficult to measure, the impact of taxation policy includes unmeasured effects of local smoke-free policies.

This study finds a significant positive income effect on consumption, which indicates that cigarettes have become more affordable. A recent study shows that between 2001 and 2016, the affordability of cigarettes in China increased 1·85 times. It is important to continue to raise the tobacco tax to offset the affordability influence on cigarette consumption.[21]

This study shows empirically that raising the tobacco tax through increasing retail prices is the most effective tobacco control policy instrument in China. Currently

For peer review only - http://bmjopen.bmj.com/site/about/guidelines.xhtml

China has a relatively low cigarette tax rate, 56% of the retail price.[16] The WHO guideline for an effective tobacco control benchmark is a tax rate of 75% of the retail price.[30] Comparing China's tax rate with the WHO guideline reveals that China has a lot of room to raise its tax on tobacco. Raising the tobacco tax will save lives, reduce smoking-related medical costs, and generate additional government revenue.

Contributorship statement

Xiaoxin Xu: Conducted literature review, participated in data collection and manuscript writing; Xiulan Zhang: Directed and verified data collection, estimated the models and drafted the findings of the models and the discussions; Teh-wei Hu: Reviewed the models and findings, participated in writing and discussions; Leonard S. Miller: Reviewed the models ad findings, participated in drafting the main findings of the models; and Mengnan Xu: Participated in data collection and verification, participated in literature review.

Competing Interest

The authors declare that they have no competing interests.

Funding

Bill & Melinda Gates Foundation

Role of the funding source

The funder had no role in the study design, collection, analysis, or interpretation of the data, writing the manuscript, or the decision to submit the paper for publication.

For peer review only

The corresponding author had full access to all data in the study and had final responsibility for the decision to submit for publication.

Data sharing statement

Extra data is available by emailing Xiulan Zhang.

Reference

1. The bill China cannot afford: Health, economic and social costs of China's tobacco epidemic. Manila: World Health Organization Regional Office for the Western Pacific, 2017.
2. Chen Z, Peto R, Zhou M, et al. Contrasting male and female trends in tobacco-attributed mortality in China: evidence from successive nationwide prospective cohort studies. *The Lancet* 2015; **386**(10002): 1447-56.
3. Development of China's tobacco industry in 2000 (in Chinese). *China Tobacco* 2001; (4).
4. Analysis of China's tobacco market in 2016 (in Chinese). *China Tobacco* 2017; (5): 62-5.
5. Yang G, Wang Y, Wu Y, Yang J, Wan X. The road to effective tobacco control in China. *The Lancet* 2015; **385**(9972): 1019-28.
6. Chen MH. Economic concerns hamper tobacco control in China. *The Lancet* 2007; **370**(9589): 729-30.
7. Clarke H, Tan BJ. Tobacco use control policies in China. *Economic Papers: A journal of applied economics and policy* 2011; **30**(4): 490-6.
8. Pratt CB. China's tobacco industry's communication practices: Paradoxes and proposals for public policymaking. *Public Relations Review* 2011; **37**(3): 318-20.
9. Huang Y. China's position in negotiating the Framework Convention on Tobacco Control and the revised International Health Regulations. *Public Health* 2014; **128**(2): 161-6.
10. Jin J. Why FCTC policies have not been implemented in China: Domestic dynamics and tobacco governance. *Journal of Health Politics, Policy and Law* 2014; **39**(3): 633-66.
11. Zheng R, Gao S, Hu T-W. Tobacco tax and tobacco control: Global experience and its application in China (in Chinese). *Finance & Trade Economics* 2013; **34**(3): 44-53.
12. Ministry of Finance, State Administration of Taxation. Notice on adjustment to excise tax on tobacco products(Taxation Document No.2009-84)(in Chinese). 2009.
13. Hu T-W, Mao Z, Shi J. Recent tobacco tax rate adjustment and its potential impact on tobacco control in China. *Tobacco Control* 2010; **19**(1): 80-2.

For peer review only - http://bmjopen.bmj.com/site/about/guidelines.xhtml

14. Shi J, Hu T-W, Mao Z. Economic effect analysis of China's tobacco excise tax reform(in Chinese). *Review on Finance and Economics* 2011; (1): 52-9.
15. Gao S, Zheng R, Hu T-W. Can increases in the cigarette tax rate be linked to cigarette retail prices? Solving mysteries related to the cigarette pricing mechanism in China. *Tobacco Control* 2012; **21**(6): 560-2.
16. Hu T-W, Zhang X, Zheng R. China has raised the tax on cigarettes: what's next? *Tobacco Control* 2015; **25**(6): 609-11.
17. Zheng R, Wang Y, Hu X. Tobacco tax: theory, system design, and policy practice (in Chinese). *Financial Minds* 2016; **1**(6): 5-30.
18. Mackay J. China: the tipping point in tobacco control. *British Medical Bulletin* 2016; **120**(1): 15-25.
19. Wedeman A. Xi Jinping's Hunt: Anti-corruption campaign or factional purge? *Modern China Studies* 2017; **24**(2): 35-94.
20. Wang L, Yan Y, Yi N, Mengwu T, Jijiang W, Yuan J. Cigarette consumer price and affordability in China: results from 2015 China adult survey. *Chinese Journal of Epidemiology* 2017; **38**(1): 69-72.
21. Zheng R, Wang Y, Hu X, Marquez PV. Cigarette affordability in China: 2001-2016. Washington, DC.: World Bank, 2017.
22. Hu T-W, Sung HY, Keeler TE. Reducing cigarette consumption in California: tobacco taxes vs an anti-smoking media campaign. *American Journal of Public Health* 1995; **85**(9): 1218-22.
23. McDowall D, McCleary R, Meidinger EE, Hay RA. Interrupted time series analysis. Thousand Oaks, CA: Sage Publications, Inc; 1980.
24. Wagner AK, Soumerai SB, Zhang F, Ross-Degnan D. Segmented regression analysis of interrupted time series studies in medication use research. *Journal of Clinical Pharmacy & Therapeutics* 2002; **27**(4): 299-309.
25. Nistal-Nuño B. Segmented regression analysis of interrupted time series data to assess outcomes of a South American road traffic alcohol policy change. *Public Health* 2017; **150**(Supplement C): 51-9.
26. Eight years' implemention of World Heath Organization Framework Convention on Tobacco Control(in Chinese). 2013. http://www.chinacdc.cn/jkzt/jkcj/sthd_3844/slhd_4153/201305/t20130515_80977.htm (accessed 12-31 2017).
27. Hu T-W, Lee AH, Mao Z. WHO Framework Convention on Tobacco Control in China: Barriers, challenges and recommendations. *Global Health Promotion* 2013; **20**(4): 13-22.
28. Xiao D, Bai C-X, Chen Z-M, Wang C. Implementation of the World Health Organization Framework Convention on Tobacco Control in China: An arduous and long-term task. *Cancer* 2015; **121**(S17): 3061-8.
29. Gao S, Zheng R. Price, tax and cigarette smoking: Simulations of China's tobacco tax policy. *Frontiers of Economics in China* 2012; **7**(4): 604-26.
30. World Health Organization. WHO technical manual on tobacco tax administration. Geneva, Switzerland: World Health Organization, 2010.

For peer review only - http://bmjopen.bmj.com/site/about/guidelines.xhtml

For peer review only

For peer review only - http://bmjopen.bmj.com/site/about/guidelines.xhtml

Figure 1: Timeline of tobacco control policies in China

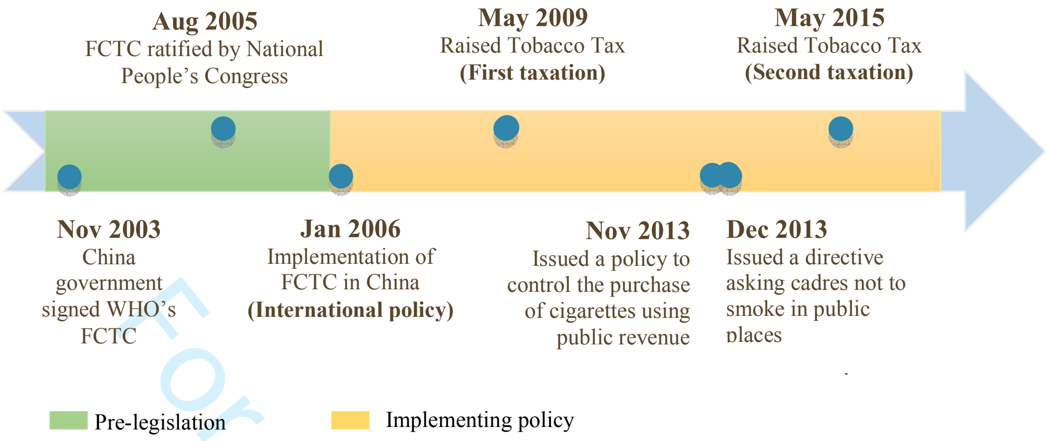

Figure 2: Estimated monthly average packs of cigarettes consumed in China per capita with and without accounting for tobacco control policies, 2000-2017

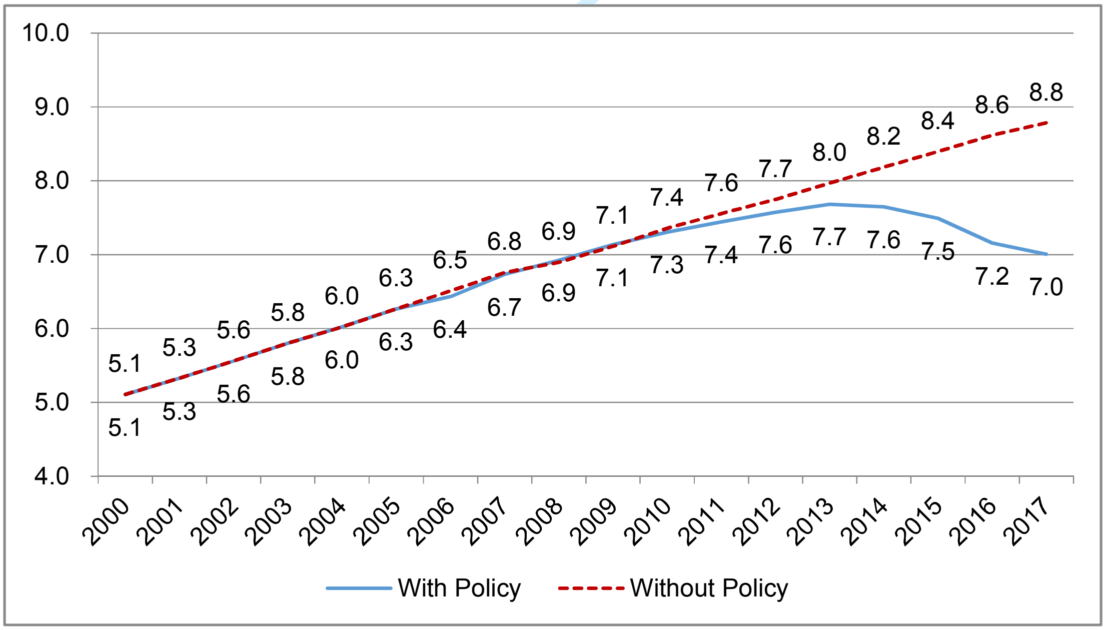

For peer review only - http://bmjopen.bmj.com/site/about/guidelines.xhtml

BMJ Open

Effects of global and domestic tobacco control policies on cigarette consumption per capita: An evaluation using monthly data in China

Journal:	*BMJ Open*
Manuscript ID	bmjopen-2018-025092.R1
Article Type:	Research
Date Submitted by the Author:	12-Feb-2019
Complete List of Authors:	Xu, Xiaoxin; Beijing Normal University, School of Social Development and Public Policy<br>Zhang, Xiulan; Beijing Normal University, School of Social Development and Public Policy<br>Hu, Teh-wei; Public Health Institute, Center for International Tobacco Control<br>Miller, Leonard; University of California, School of Social Welfare<br>XU, Mengnan; Beijing Normal University, School of Social Development and Public Policy
**Primary Subject Heading**:	Smoking and tobacco
Secondary Subject Heading:	Global health, Health economics, Health policy
Keywords:	global health, smoking, global and domestic health policies, China, tobacco control

SCHOLARONE™
Manuscripts

Effects of global and domestic tobacco control policies on cigarette consumption per capita: An evaluation using monthly data in China

Word Count: 3860

Xu Xiaoxin, Xiulan Zhang, Teh-wei Hu, Leonard Miller, Mengnan Xu

Authors

Xiaoxin Xu, Ph.D.,

School of Social Development and Public Policy,

Beijing Normal University, China

Email: xuxiaoxin@bnu.edu.cn

Xiulan Zhang, Ph.D.,

School of Social Development and Public Policy,

Beijing Normal University, China

Email: zhang99@bnu.edu.cn

Teh-wei Hu, Ph.D.,

Center for International Tobacco Control,

Public Health Institute, Oakland, CA, USA

Email: thu@berkeley.edu

Leonard S. Miller, Ph.D.,

School of Social Welfare,

For peer review only

University of California, Berkeley, CA, USA

Email: lsmiller2331@gmail.com

Mengnan Xu, B.S.,

School of Social Development and Public Policy,

Beijing Normal University, China

Email: pumpkinggg@126.com

Correspondence: Xiulan Zhang,

School of Social Development and Public Policy, Beijing Normal University, Beijing, China, 19 Xinjiekouwai Street, Haidian District, Beijing, 100875, China. Tel: (86-10) 5880-1512; Fax: (86-10) 5880-0366; Email: zhang99@bnu.edu.cn

For peer review only

For peer review only - http://bmjopen.bmj.com/site/about/guidelines.xhtml

Abstract

Introduction China consumes 44% of the world's cigarettes. Robust tobacco control measures are needed to stop the continuing trend of increased cigarette consumption. This paper looks into the effective policies that might have the desired impact on reducing tobacco use in China.

Methods Monthly data on cigarette consumption per capita from January 2000 to June 2017, calculated from China National Tobacco Corporation's monthly sales data and China National Bureau of Statistics' demographic data, are used to estimate the impact of specific policies on China's tobacco consumption. The policies studied include the World Health Organization's (WHO) Framework Convention on Tobacco Control (FCTC), national tobacco-related policy changes, and two tobacco tax increases implemented in China during the study period. Segmented regression analysis is used to estimate the immediate effect of the policies studied and changes in the time trends resulting from these policy changes.

Findings The impact of national policy changes in China is almost 20 times greater than the impact of the WHO's FCTC treaty itself, and national policy changes in tobacco control are a determining factor in reversing the trend of increased tobacco consumption in China. The 2015 tax increase, which raised retail cigarette prices, produced both immediate and trend effects, with a total marginal effect 7.8 times that of the 2009 tax increase, which did not result in higher cigarette prices for the consumer.

Interpretations Translating global social norms of tobacco control into national policies will generate much higher impact on average cigarette consumption, and tobacco taxes

For peer review only - http://bmjopen.bmj.com/site/about/guidelines.xhtml

that are reflected in the retail prices will be more effective in reducing the consumption of cigarettes.

Strengths and limitations of this study

1. To the best of our knowledge, this study is the first systematic evaluation of the impact of both domestic and global tobacco control policies on tobacco consumption in China.
2. The study compares the effectiveness of the global FCTC and domestic policies to reduce cigarette consumption in China over a period of 17.5 years.
3. The data used for the policy evaluation covers the periods from no tobacco control policies in China to the implementation of FCTC policies to the changed national policies to the specific tax increases enacted in China in 2009 and 2015.
4. Using the interrupted time series model, the study not only examines the immediate impact of each policy on tobacco consumption, but also the policy impact on tobacco consumption trends.
5. The limitations of this study are that the social norm change has not been incorporated into the models, and the cigarette consumption is based on wholesales rather than retails.

For peer review only

Introduction

The 315 million smokers in China consume 44% of the world's cigarettes, and their average consumption is 2.3 times the world average.[1] Tobacco use increases medical expenses by billions.[1] Each year, one million people in China, many of them young, die of tobacco-related diseases.[2] China's rapid economic development in the past 40 years has been accompanied by significant growth in the country's total cigarette consumption. In 2000, the China National Tobacco Corporation (CNTC), the state-owned tobacco monopoly, sold 76.92 billion packs of cigarettes;[3] by 2014, the number had grown to 127.48 billion packs,[4] an increase of 65.8 %.

China signed the World Health Organization's (WHO) Framework Convention on Tobacco Control (FCTC) in 2003; the China National People's Congress (CNPC) ratified the treaty in 2005, and China began implementing the FCTC in 2006, indicating China government should fulfill its legal obligation in accordance with the treaty.[5] The ratifying and implementation of the FCTC provided a moral and legal high ground for advocates on tobacco control[6], though there was still a long way for the implementation of specific articles.[7] In this paper, we refer to the WHO framework as an international policy. While the Chinese government has made some effort to control tobacco use, strong interference from the China State Tobacco Monopoly Administration (STMA), which owns CNTC, in favoring economic concerns over social concerns, has led to slow development and implementation of the full tobacco control policy measures. [8-13]

Between 2006 and 2015, China increased tobacco taxes twice. The first tax increase was introduced in May 2009 and was not reflected in cigarette retail prices directly. As a result, the increase had minimal immediate impact on consumers, but it might have more

For peer review only - http://bmjopen.bmj.com/site/about/guidelines.xhtml

long term impact by changing cigarette product structure and consequent average price raising.[14] The 2009 adjustment raised the ad valorem tax from 45% to 56% at the producer price level for class A cigarettes and from 30% to 36% for class B cigarettes. The new policy also introduced a new 5% ad valorem tax at the wholesale price level.[15 16] The intent of this 2009 adjustment was to raise government revenue from CNTC, China's tobacco producer, not to serve as a tobacco control policy instrument. Under the new scheme, the government forbade changes in the retail prices of cigarettes.[16-18] The policy was introduced primarily to counteract the impact of the global financial crisis on government revenue. Before this tax increase in May 2009, China's public revenue had declined for the previous seven months. Between January and April 2009, public revenue decreased 9.9% while public spending increased 31.7%. The financial pressure prompted the government to raise the tobacco tax. In other words, this policy was driven by an economic goal, and because the policy forbade the tobacco industry from adding the tax increase to the retail price of cigarettes, the social goal was not considered at all.[16 17] This is possible because cigarette pricing mechanism is unique in China under its tobacco monopoly system. Both the cigarette allocation price, the price at which the tobacco producers offer cigarettes to the wholesalers, and wholesale price, the price at which the wholesalers offer cigarettes to retailors, are controlled by China State Tobacco Monopoly Administration (STMA). In 2009 tobacco tax adjustment, STMA reduced the wholesale profit margin but maintained the retail price unchanged. In this sense, the 2009 tobacco tax adjustment could be regarded as a profit tax adjustment rather than an excise tax adjustment. The second tobacco tax increase occurred in May 2015. Unlike the 2009 tax adjustment, the 2015 adjustment moved the increase from the tax base at the wholesale

For peer review only - http://bmjopen.bmj.com/site/about/guidelines.xhtml

price level to the retail price level, a significant step away from the 2009 increase and toward China's tobacco control agenda.[19] The 2015 tax increase initiated a 0.10 RMB tax per pack at the wholesale price level and increased the ad valorem tax from 5% to 11%, a 6% point increase also at the wholesale price level. However, this time, the Chinese government allowed the tobacco industry to shift this new tax increase to the retail price, an estimated 10% increase in the retail price of cigarettes.[20]

China's new administration came into power in 2013. Support from its top leader, President Xi, began to change the policy direction of tobacco control in China.[21] The national policy change began with the anti-corruption campaign, which was aimed at the problem of corruption within the party, state, and business sectors.[22] In November 2013, the government forbade the purchase of cigarettes using public revenue. A month later additional policies were announced that prohibited state employees/officers from smoking in public places. This significant policy initiative can be considered a major government effort to change the social norm of smoking habits in China. Figure 1 shows the timeline of major tobacco control policies in China.

Insert Figure 1 Here

One factor that has influenced the trend of increased cigarette consumption in China is the rapid growth of personal income. China has experienced the largest economic transformation in human history. Based on data from China National Bureau of Statistics (http://data.stats.gov.cn/ks.htm?cn=C01&zb=A0501), following the 1978 economic reform, the Chinese economy grew around 9.5% each year, becoming the second largest

For peer review only

For peer review only - http://bmjopen.bmj.com/site/about/guidelines.xhtml

economy in the world according to World Bank's report.[23] In recent years, China's economic performance has remained at a relatively high level of 7% growth. While the income of people in China also has increased significantly, an increase in cigarette prices has not accompanied the GDP growth, thus making cigarettes more affordable over time.[24 25]

Waiting for China to take robust measures to control its tobacco use, change the social norm and policy landscape, reduce the institutional barriers created by the China STMA, and counteract the increased consumption of cigarettes resulting from income growth is a long and frustrating process.

The purpose of this paper is to estimate the relative impacts of four tobacco control policy interventions on tobacco consumption in China: first ever international public health treaty WHO's FCTC, the government's 2013 national policy forbidding use of general revenue to purchase cigarettes and smoking in public by state employees/officers, and the tax increases of 2009 and 2015. This is the first study to estimate the combined impact of international and domestic tobacco control policy changes on long-term trends in tobacco consumption in China.

Methods

Data

We used the monthly data on cigarette sales from January 2000 to June 2017, a total of 210 months of data reported by CNTC. Sales data, collected by CNTC, are based on the purchases of retailers, so the exact monthly sales are determined by the dates when retailers buy from cigarette distributors. We extracted these data from *China Tobacco Magazine* and its website (www.echinatobacco.com), hosted by CNTC.

For peer review only - http://bmjopen.bmj.com/site/about/guidelines.xhtml

The population data were based on China Statistical Yearbook, extracted from the website of China National Bureau of Statistics (http://www.stats.gov.cn/tjsj/ndsj/#). Between 2000 and 2016, China's total population increased by 9.1%. To adjust for the effect of population growth on cigarette sales, this study uses the average packs of cigarettes consumed each month per capita.[26] To estimate policy impacts, we include the GDP growth rate, the timing of policy interventions, and trends initiated by each of the four policy interventions studied here.

During the study period, several tobacco control policies were implemented in China. As discussed in the introduction section, the first was the ratification of WHO's FCTC, the implementation of which began in January 2006. In May 2009, China raised cigarette taxes, but the increase was not reflected in the retail price. In 2013, tobacco control received top leadership support. In November, a national policy was issued forbidding government funds from being used to purchase cigarettes for officials, and a month later, in December, the Central Committee of the Communist Party of China (CCCPC) and the State Council jointly issued a policy prohibiting cadres from smoking in public places. This national policy targeted party officials and government agencies. In May 2015, China again raised cigarette taxes, this time allowing retail prices to rise.

Since the implementation of WHO's FCTC, smoke-free policies have been established in different cities or regions of China. The Beijing Municipal Government passed the strictest smoke-free regulation in May 2015. But a national smoke-free law has not passed. Therefore, the effect of smoke-free policies is an unmeasured effect in the model.

We divided the analysis into five time periods: (1) before the FCTC was implemented (January 2000 to December 2005); (2) between implementation of the FCTC and the first

For peer review only - http://bmjopen.bmj.com/site/about/guidelines.xhtml

tax policy adjustment (January 2006 to April 2009); (3) between the first tax policy adjustment and implementation of the national policy change (May 2009 to October 2013); (4) between implementation of the national policy change and the second tax policy adjustment (November 2013 to April 2015); and (5) the period after all policies were implemented (May 2015 to June 2017).

Table 1 presents descriptive statistics of the GDP growth rates and average packs of cigarettes consumed during each of the five periods studied.

Table 1 Average cigarette consumption and GDP growth in different periods

Period	Policies	Number of Months	Average Consumption (Pack/month/per capita)	Average GDP Growth (%)
Jan 2000 to Dec 2005	No Policies	72	5.66 (0.57)	9.54 (1.06)
Jan 2006 to Apr 2009	FCTC Only	40	6.78 (1.22)	11.86 (2.02)
May 2009 to Oct 2013	FCTC/Tax1	54	7.43 (1.83)	8.87 (1.11)
Nov 2013 to Apr 2015	FCTC/Tax1/National	18	7.69 (2.14)	7.32 (0.25)
May 2015 to Jun 2017	All Policies	26	7.20 (1.56)	6.81 (0.10)

The GDP growth rates during the 17 years for which we have data reached 14.16% in 2007 and then dropped to 6.7% in 2016. The average growth rate over the analysis period was 9.36%. The decline in GDP growth rates began in 2012.

Patient and Public Involvement

There is no patient and public involvement of the data collection.

Statistical Analysis

Segmented regression analysis of interrupted time series is an effective statistical method to evaluate longitudinal effects of time-delimited interventions,[27] and it is widely used in assessing policy impact especially where randomization is not feasible.[28] In this model, two parameters are estimated for each intervention studied: level and trend. The level

For peer review only - http://bmjopen.bmj.com/site/about/guidelines.xhtml

parameter defines the y-intercept, which is the immediate effect of the intervention on the outcome. The time trend interaction with the intervention variable is the rate of change (the slope), which measures the gradual change of the outcomes due to the intervention.[29 30]

We estimated the segmented regression model using SAS AUTOREG procedures to assess the longitudinal impact of tobacco control policies on the average cigarette consumption per month per capita. We estimated levels and trends of the four interventions: FCTC (2005), first taxation (2009), national policies (2013), and second taxation (2015). The monthly pattern of sales was adjusted by the AR parameters in AUTOREG procedure.

Results

Table 2 presents an estimation of the model describing average sales of packs of cigarettes consumed per person per month. Overall, the model is very significant with a total R-squared of 0.9416. The transformed R-squared is 0.995, indicating an extremely high fit of the model and the existence of autocorrelation.

Table 2 Auto-regression model estimate of the per-capita monthly cigarette consumption

Maximum-Likelihood Estimates of the Model			
SSE	30.94	DFE	187
MSE	0.165	Root MSE	0.407
SBC	338.86	AIC	261.88
MAE	0.27	AICC	267.81
MAPE	4.04	HQC	293.00
Log Likelihood	-107.94	Observations	210
Total R^2			0.9416
Transformed R^2			0.9950
**Parameter Estimates**			
Variable	Estimate	t Value	Pr > |t|
Intercept	4.8353	70.45	<.0001
GDP Growth	0.0180	2.10	0.0371
Time	0.0184	32.99	<.0001

For peer review only - http://bmjopen.bmj.com/site/about/guidelines.xhtml

FCTC	-0.1101	-2.53	0.0121
FCTC_time	0.0046	2.37	0.0189
Tax1	-0.0462	-1.38	0.1686
Tax1_time	-0.0097	-7.55	<.0001
National	-0.3056	-5.63	<.0001
National_time	0.0078	1.72	0.0871
Tax2	-0.4309	-6.39	<.0001
Tax2_time	-0.0382	-8.30	<.0001
AR1	0.6277	8.87	<.0001
AR2	0.5907	8.04	<.0001
AR3	0.5834	7.83	<.0001
AR4	0.5588	7.33	<.0001
AR5	0.5435	7.00	<.0001
AR6	0.5458	6.85	<.0001
AR7	0.5412	6.72	<.0001
AR8	0.5397	6.72	<.0001
AR9	0.5546	6.98	<.0001
AR10	0.5662	7.23	<.0001
AR11	0.5644	7.32	<.0001
AR12	-0.3572	-4.83	<.0001

The time effect is positive and significant, indicating the urgency to interrupt the trend of increasing cigarette consumption in China to reduce the burden of diseases and death due to smoking.

The parameter estimate shows that GDP growth has a positive and statistically significant impact on monthly cigarette consumption. GDP growth indicates a macro income effect; this effect conforms with the literature that the income elasticity of cigarette consumption is positive.

In terms of the impact of tobacco control policies, implementation of the FCTC in 2006 resulted in an immediate reduction in the number of cigarettes consumed. However, after the initial reduction, consumption again rose over time, indicating that either the tobacco industry developed new strategies to counteract the FCTC policy or consumers resumed the intensity of their smoking habits after the initial reaction to the macro-policy change. Similar to what happened after implementation of the international treaty, when the CCCPC and the State Council jointly issued a national policy on cadres and governments

For peer review only - http://bmjopen.bmj.com/site/about/guidelines.xhtml

in 2013, consumption of cigarettes dropped immediately. The drop following announcement of the national policy was about three times the drop in consumption after implementation of the FCTC. Again, similar to what happened after implementation of the FCTC, the trend after the change in national policy was positive subsequent to the drop, but not statistically significant. This finding shows that while the 2013 national policy changes aimed at changing cigarette-related social norms could immediately impact average sales, the after effect was counteracted by either consumer habits or more aggressive marketing strategies by the tobacco industry.

As for China's two tobacco tax initiatives, the coefficient of the Tax 1 (2009) variable is not statistically significant, as one would have expected. Over time, the tobacco industry restructured its market share but the magnitude of the coefficient of the time and tax interaction term is still very small, though statistically significant. However, the coefficient of the Tax 2 (2015) variable and its time trend interaction term are both statistically significant with a magnitude four times larger than the FCTC effect and 40 percent higher than the national policy effect. As shown from the coefficient and its interaction term, the 2015 tobacco tax increase (Tax 2) essentially reduced per capita monthly consumption by 0.43 pack initially and then continued to reduce consumption by 0.04 pack per capita per month over time.

The implementation of both tax increases (2009 and 2015) resulted in similar initial effects and time trends. The initial effect of the second (2015) tax increase, aimed at wholesale and retail prices, was about ten times the initial effect of the 2009 tax increase. The trend effect of the 2015 policy was about four times the trend effect of the first tax

For peer review only - http://bmjopen.bmj.com/site/about/guidelines.xhtml

increase. In addition, unmeasured smoke-free model effects might have contributed to the big impact of the 2015 tax increase.

This finding indicates that unless specific policies are targeted at smokers, generalized policies advocating tobacco control may result in some immediate effects, but they won't be able to change the consumption of tobacco over time.

Tax increases are much more effective at changing tobacco consumption than generalized policies. This was true of even the first tax increase (in 2009), which was not reflected in the retail price of cigarettes. When the tax increase was factored into the retail price (in 2015), the impact on cigarette consumption was much larger and was sustained over time.

Figure 2 presents China's average monthly packs of cigarettes consumed per capita per year from 2000-2017 with and without accounting for tobacco control policies. Before the FCTC policy was implemented, between 2000 and 2005, average consumption increased from 5.1 packs to 6.3 packs per capita per month, an increase of 23.5% in six years.

Between 2006 and 2013, monthly cigarette consumption grew from 6.4 packs to 7.7 packs, an increase of 20.3% in seven years. Consumption then began to decrease in 2013. By the end of 2016, the average number of packs of cigarettes consumed monthly had dropped from 7.7 to 7.2, a 6.5% decrease in three years. Without the tax increase, average consumption was predicted at 8.6 packs, 16.3% higher than with the tax increase.

The 2013 policy announcements by the Chinese national government changed smoking-related social norms. Combined with the global FCTC intervention and the first tax increase (2009), the growing trend of cigarette consumption per month per capita in

For peer review only - http://bmjopen.bmj.com/site/about/guidelines.xhtml

China finally began to decline, and the second tax increase (2015) had a much bigger impact on the downward trend.

Insert Figure 2 Here.

Based on the model estimates, we calculated the total impact of various policies on the average number of packs of cigarettes consumed per month. Table 3 presents the percentage change in average monthly consumption of cigarettes, with and without tobacco control policies. The percentage effect is calculated as follows:

$$\% \text{ Change} = (Y\hat{}_{policies=1} - Y\hat{}_{policies=0})/Y\hat{}_{policies=0}$$

Table 3 Impact of tobacco control policies on average monthly cigarette consumption per capita

Policy Period	Predicted Monthly Consumption in Packs of Cigarettes with Policies ($Y\hat{}_{policies=1}$)	Predicted Monthly Consumption in Packs of Cigarettes without Policies ($Y\hat{}_{policies=0}$)	% Change ($Y\hat{}_{policies=1}$ - $Y\hat{}_{policies=0}$) /$Y\hat{}_{policies=0}$	Incremental Change
No Policies	5.680	5.680	0.00%	0.00%
FCTC Only	6.737	6.754	-0.25%	-0.25%
FCTC/Tax1	7.450	7.566	-1.54%	-1.29%
FCTC/Tax1/National	7.668	8.202	-6.51%	-4.97%
FCTC/Tax1/National/Tax2	7.176	8.598	-16.54%	-10.03%

Table 3 shows that during the 40-month period when only the FCTC policy was in effect (January 2006 through April 2009), average cigarette consumption dropped 0.25%, due mainly to the initial impact of the FCTC.

During the 54-month period that includes implementation of the FCTC and the first tax increase (May 2009 through October 2013), average consumption dropped 1.54%, and

For peer review only - http://bmjopen.bmj.com/site/about/guidelines.xhtml

the marginal effect of the 2009 tax increase was -1.29%, indicating a very limited effect when the tax increase was not factored into the retail price.

During the 18-month period following issuance of the national policies (November 2013 through April 2015), but prior to the second tax increase instituted in May 2015, the average consumption of cigarettes dropped 6.51%, and the national policy changes alone reduced monthly consumption by 4.97%.

After the second tax increase announced in May 2015, a big decline occurred in cigarette consumption. In the 26-month period following this tax increase (May 2015 through June 2017), the average consumption of cigarettes dropped 16.54%, due mainly to the effect of the second tax increase, which alone brought down average monthly consumption by 10.03%.

Table 4 presents the predicted effects of the four policies studied on cigarette consumption measured in million packs.

Table 4 Impact of tobacco control policies on cigarettes consumption in million packs

Period	FCTC	1st Taxation	National	2nd Taxation	All Policies
Jan 2006 to Apr 2009	893	0	0	0	893
May 2009 to Oct 2013	1,377	7,106	0	0	8,484
Nov 2013 to Apr 2015	505	2,606	10,042	0	13,153
May 2015 to Jun 2017	771	3,980	15,336	30,949	51,036
Total	3,547	13,693	25,377	30,949	73,567

Between January 2006 and June 2017, China consumed 1.348 trillion packs of cigarettes. The reduction in total consumption attributable to the policy changes was 73.6 billion packs, which is 5.18% of the sales predicted without policy interventions.

Implementation of the FCTC decreased consumption by 3.5 billion packs, the first tax

For peer review only - http://bmjopen.bmj.com/site/about/guidelines.xhtml

increase (2009) reduced consumption by 13.7 billion, the national policy announcements decreased it by 25.4 billion packs, and the largest reduction came from the second tax increase (2015), including unmeasured local smoke-free policies--almost 31 billion packs in just 26 months.

From announcement of the 2013 national policy change through June 2017, China reduced the sales of cigarettes by 64.2 billion packs, a 12.57% reduction in the average consumption of cigarettes in China.

Discussion

The World Health Organization's FCTC, an international treaty, aims to provide a roadmap to address the global tobacco epidemic using effective measures and strategies. China ratified the treaty in November 2005 and began implementation in January 2006. Calculated from Table 3, this study finds that the impact of national policy changes has been almost 20 times larger than the impact of the WHO's FCTC treaty itself, and that national tobacco control policy changes in China have been a determining factor in reversing the increasing trend of tobacco consumption. In other words, implementing an international treaty requires national policy and social norm changes to achieve the goal of reducing tobacco consumption. Ratification of the treaty alone without domestic policy implementation will have a very minimal effect.

The process of integrating global social norms with domestic policy change took exactly 10 years in China (November 2003 to November 2013).[31] Our study finds that after the immediate effects of the policy changes were noted, the powerful STMA developed countermeasures to dilute the impact of the policy changes. This finding confirms the

For peer review only - http://bmjopen.bmj.com/site/about/guidelines.xhtml

challenges faced by and the persistence required for the global and national tobacco control communities. [7,32]

Between 2006 and 2013, although the government raised the tobacco tax in May 2009, the economic goal of increasing government revenue overpowered the social goal of reducing tobacco consumption. The tax increase did not result in higher cigarette prices for the consumer, thus minimizing the impact of this policy on consumption.[17,33]

When the 2015 tax policy raised retail cigarette prices, both immediate and trend effects were very significant, and the total marginal effect was 7.8 times that of the 2009 tax increase (the ratio is calculated as the marginal effect of tax2 which is the difference of FCTC/Tax1/National/Tax2 and FCTC/Tax1/National presented in table 3: -16.54% and -6.51%=-10.03%, and the marginal effect of tax1 is the difference of FCTC/Tax1 and FCTC Only: -1.54% and -0.25%=-1.29%. The ratio of marginal effect tax2 and tax1 is -10.03% and -1.29%=7.8). This finding indicates that tobacco control policies should be more robust and target consumers more directly through higher prices and tougher smoke-free regulations. Because China has no national smoke-free law, and the impact of various local smoke-free regulations on national cigarette consumption is difficult to measure, the impact of taxation policy includes unmeasured effects of local smoke-free policies.

This study finds a significant positive income effect on consumption, which indicates that cigarettes have become more affordable. A recent study shows that between 2001 and 2016, the affordability of cigarettes in China increased 1.85 times. It is important to continue to raise the tobacco tax to offset the affordability influence on cigarette consumption.[25]

For peer review only - http://bmjopen.bmj.com/site/about/guidelines.xhtml

This study shows empirically that raising the tobacco tax through increasing retail prices is the most effective tobacco control policy instrument among the few policies implemented in China. Currently China has a relatively low cigarette tax rate, 56% of the retail price.[19] The WHO guideline for an effective tobacco control benchmark is a tax rate of 75% of the retail price.[34] Comparing China's tax rate with the WHO guideline reveals that China has a lot of room to raise its tax on tobacco. Raising the price of cigarettes will save lives, reduce smoking-related medical costs, and generate additional government revenue.

This study has some limitations. During the study period, social norm of smoking has changed significantly, because of the tobacco control policy changes, and increase of the health awareness toward smoking. This change of social norm has not been incorporated into the model estimates. The consumption of cigarette data are based on wholesales rather than retail data.

Contributorship statement

Xiaoxin Xu: Conducted literature review, participated in data collection and manuscript writing; Xiulan Zhang: Directed and verified data collection, estimated the models and drafted the findings of the models and the discussions; Teh-wei Hu: Reviewed the models and findings, participated in writing and discussions; Leonard S. Miller: Reviewed the models ad findings, participated in drafting the main findings of the models; and Mengnan Xu: Participated in data collection and verification, participated in literature review.

Competing Interest

For peer review only - http://bmjopen.bmj.com/site/about/guidelines.xhtml

The authors declare that they have no competing interests.

Funding

Bill & Melinda Gates Foundation

Role of the funding source

The funder had no role in the study design, collection, analysis, or interpretation of the data, writing the manuscript, or the decision to submit the paper for publication. The corresponding author had full access to all data in the study and had final responsibility for the decision to submit for publication.

Data sharing statement

Extra data is available by emailing Xiulan Zhang.

References

1. The Bill China Cannot Afford: Health, Economic and Social Costs of China's Tobacco Epidemic. Manila: World Health Organization Regional Office for the Western Pacific, 2017.
2. Chen Z, Peto R, Zhou M, et al. Contrasting male and female trends in tobacco-attributed mortality in China: evidence from successive nationwide prospective cohort studies. *The Lancet* 2015;386(10002):1447-56. doi: https://doi.org/10.1016/S0140-6736(15)00340-2
3. Development of China's Tobacco Industry in 2000 (in Chinese). *China Tobacco* 2001(4)
4. Analysis of China's Tobacco Market in 2016 (in Chinese). *China Tobacco* 2017(5):62-65.
5. Yang G. Introduction: China and the Negotiation of WHO FCTC. In: Yang G, ed. Tobacco control in China. Singapore: Springer 2018:1-16
6. Huang J. Public interest litigation and tobacco control in China. In: Yang G, ed. Tobacco control in China. Singapore: Springer 2018:119-40
7. Xiao D, Bai C-X, Chen Z-M, et al. Implementation of the World Health Organization Framework Convention on Tobacco Control in China: An arduous and long-term task. *Cancer* 2015;121(S17):3061-68. doi: 10.1002/cncr.29608
8. Yang G, Wang Y, Wu Y, et al. The road to effective tobacco control in China. *The Lancet* 2015;385(9972):1019-28. doi: https://doi.org/10.1016/S0140-6736(15)60174-X

For peer review only - http://bmjopen.bmj.com/site/about/guidelines.xhtml

For peer review only

9. Chen MH. Economic concerns hamper tobacco control in China. *The Lancet* 2007;370(9589):729-30. doi: 10.1016/S0140-6736(07)61359-2
10. Clarke H, Tan BJ. Tobacco Use Control Policies in China. *Economic Papers: A journal of applied economics and policy* 2011;30(4):490-96. doi: 10.1111/j.1759-3441.2011.00132.x
11. Pratt CB. China's tobacco industry's communication practices: Paradoxes and proposals for public policymaking. *Public Relations Review* 2011;37(3):318-20. doi: https://doi.org/10.1016/j.pubrev.2011.03.010
12. Huang Y. China's position in negotiating the Framework Convention on Tobacco Control and the revised International Health Regulations. *Public Health* 2014;128(2):161-66. doi: https://doi.org/10.1016/j.puhe.2013.08.003
13. Jin J. Why FCTC Policies Have Not Been Implemented in China: Domestic Dynamics and Tobacco Governance. *Journal of Health Politics, Policy and Law* 2014;39(3):633-66. doi: 10.1215/03616878-2682630
14. Zheng R, Gao S, Hu T-W. Tobacco Tax and Tobacco Control: Global Experience and Its application in China (in Chinese). *Finance & Trade Economics* 2013;34(3):44-53.
15. Ministry of Finance, State Administration of Taxation. Notice on Adjustment to Excise Tax on Tobacco Products(Taxation Document No.2009-84)(in Chinese), 2009.
16. Hu T-w, Mao Z, Shi J. Recent tobacco tax rate adjustment and its potential impact on tobacco control in China. *Tobacco Control* 2010;19(1):80-82.
17. Shi J, Hu T-w, Mao Z. Economic Effect Analysis of China's Tobacco Excise Tax Reform(in Chinese). *Review on Finance and Economics* 2011(1):52-59.
18. Gao S, Zheng R, Hu T-w. Can increases in the cigarette tax rate be linked to cigarette retail prices? Solving mysteries related to the cigarette pricing mechanism in China. *Tobacco Control* 2012;21(6):560-62.
19. Hu T-w, Zhang X, Zheng R. China has raised the tax on cigarettes: what's next? *Tobacco Control* 2015;25(6):609-11.
20. Zheng R, Wang Y, Hu X. Tobacco tax: theory, system design, and policy practice (in Chinese). *Financial Minds* 2016;1(6):5-30.
21. Mackay J. China: the tipping point in tobacco control. *British Medical Bulletin* 2016;120(1):15-25. doi: 10.1093/bmb/ldw043
22. Wedeman A. Xi Jinping's Hunt: Anti-Corruption Campaign or Factional Purge? *Modern China Studies* 2017;24(2):35-94.
23. World Bank. World Development Report 2012 : Gender Equality and Development. Washington, DC: The World Bank Group, 2012.
24. Wang L, Yan Y, Yi N, et al. Cigarette consumer price and affordability in China: results from 2015 China adult survey. *Chinese Journal of Epidemiology* 2017;38(1):69-72.
25. Zheng R, Wang Y, Hu X, et al. Cigarette Affordability in China: 2001-2016. Washington, DC.: World Bank, 2017.
26. Hu TW, Sung HY, Keeler TE. Reducing cigarette consumption in California: tobacco taxes vs an anti-smoking media campaign. *American Journal of Public Health* 1995;85(9):1218-22. doi: 10.2105/ajph.85.9.1218

For peer review only

27. McDowall D, McCleary R, Meidinger EE, et al. Interrupted Time Series Analysis. Thousand Oaks, CA: Sage Publications, Inc 1980.
28. Wagner AK, Soumerai SB, Zhang F, et al. Segmented regression analysis of interrupted time series studies in medication use research. *Journal of Clinical Pharmacy and Therapeutics* 2002;27(4):299-309. doi: doi:10.1046/j.1365-2710.2002.00430.x
29. Wagner AK, Soumerai SB, Zhang F, et al. Segmented regression analysis of interrupted time series studies in medication use research. *Journal of Clinical Pharmacy & Therapeutics* 2002;27(4):299-309.
30. Nistal-Nuño B. Segmented regression analysis of interrupted time series data to assess outcomes of a South American road traffic alcohol policy change. *Public Health* 2017;150(Supplement C):51-59. doi: https://doi.org/10.1016/j.puhe.2017.04.025
31. Eight years' implemention of World Heath Organization Framework Convention on Tobacco Control(in Chinese) 2013 [Available from: http://www.chinacdc.cn/jkzt/jkcj/sthd_3844/slhd_4153/201305/t20130515_80977.htm accessed 12-31 2017.
32. Hu T-W, Lee AH, Mao Z. WHO Framework Convention on Tobacco Control in China: barriers, challenges and recommendations. *Global Health Promotion* 2013;20(4):13-22. doi: 10.1177/1757975913501910
33. Gao S, Zheng R. Price, Tax and Cigarette Smoking: Simulations of China's Tobacco Tax Policy. *Frontiers of Economics in China* 2012;7(4):604-26. doi: doi:https://doi.org/10.3868/s060-001-012-0028-2
34. World Health Organization. WHO Technical Manual on Tobacco Tax Administration. Geneva, Switzerland: World Health Organization, 2010.

For peer review only

For peer review only - http://bmjopen.bmj.com/site/about/guidelines.xhtml

Figure 1 Timeline of tobacco control policies in China

For peer review only

For peer review only - http://bmjopen.bmj.com/site/about/guidelines.xhtml

Figure 2 Estimated monthly average packs of cigarettes consumed in China per capita with and without accounting for tobacco control policies, 2000-2017. Dotted red line (without policy), trend without tobacco control policies; Blue line(with policy), trend with tobacco control policies. Both lines are predicted values from the time-series model parameters.

For peer review only

For peer review only - http://bmjopen.bmj.com/site/about/guidelines.xhtml

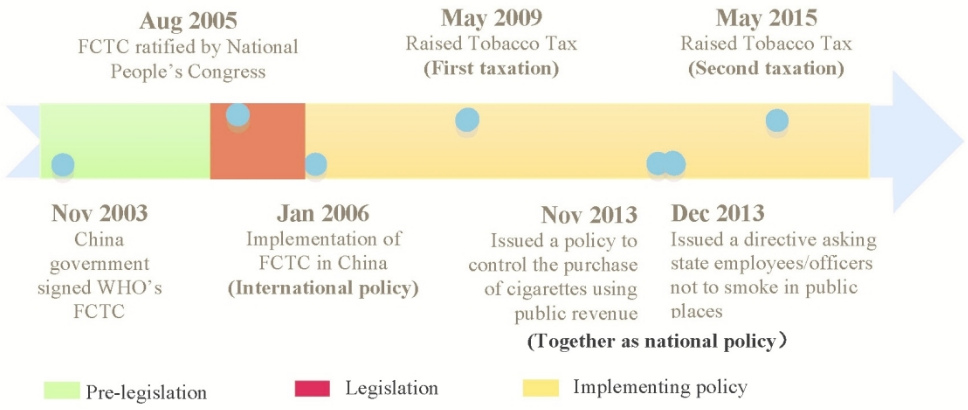

Figure 1 Timeline of tobacco control policies in China

461x196mm (96 x 96 DPI)

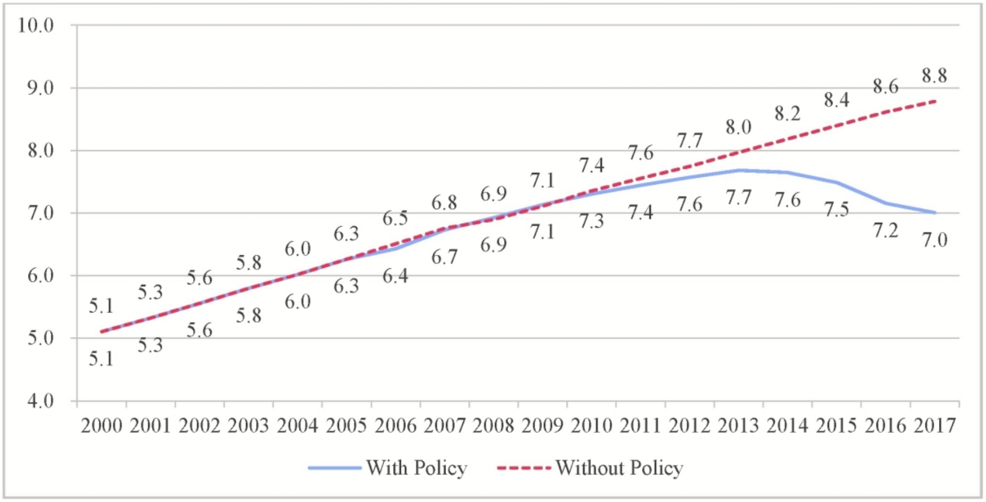

Figure 2 Estimated monthly average packs of cigarettes consumed in China per capita with and without accounting for tobacco control policies, 2000-2017. Dotted red line (without policy), trend without tobacco control policies; Blue line(with policy), trend with tobacco control policies. Both lines are predicted values from the time-series model parameters.

546x284mm (96 x 96 DPI)

STROBE Statement—checklist of items that should be included in reports of observational studies
(Shown in form of a/b, a means page number, and b means line number; n/a means not applicable.)

	Item No	Recommendation
**Title and abstract**	1	(*a*) Indicate the study's design with a commonly used term in the title or the abstract (1/4-7, 3/15-33)
		(*b*) Provide in the abstract an informative and balanced summary of what was done and what was found(3/35-52&4/3-6)
**Introduction**		
Background/rationale	2	Explain the scientific background and rationale for the investigation being reported(6/24-8/22)
Objectives	3	State specific objectives, including any prespecified hypotheses(8/24-38)
**Methods**		
Study design	4	Present key elements of study design early in the paper (n/a)
Setting	5	Describe the setting, locations, and relevant dates, including periods of recruitment, exposure, follow-up, and data collection(n/a)
Participants	6	(*a*) *Cohort study*—Give the eligibility criteria, and the sources and methods of selection of participants. Describe methods of follow-up(n/a)<br>*Case-control study*—Give the eligibility criteria, and the sources and methods of case ascertainment and control selection. Give the rationale for the choice of cases and controls(n/a)<br>*Cross-sectional study*—Give the eligibility criteria, and the sources and methods of selection of participants(n/a)
		(*b*) *Cohort study*—For matched studies, give matching criteria and number of exposed and unexposed(n/a)<br>*Case-control study*—For matched studies, give matching criteria and the number of controls per case(n/a)
Variables	7	Clearly define all outcomes, exposures, predictors, potential confounders, and effect modifiers. Give diagnostic criteria, if applicable(8/45-10/39)
Data sources/ measurement	8*	For each variable of interest, give sources of data and details of methods of assessment (measurement). Describe comparability of assessment methods if there is more than one group(8/45-10/13)
Bias	9	Describe any efforts to address potential sources of bias(n/a)
Study size	10	Explain how the study size was arrived at(n/a)
Quantitative variables	11	Explain how quantitative variables were handled in the analyses. If applicable, describe which groupings were chosen and why(8/45-9/17)
Statistical methods	12	(*a*) Describe all statistical methods, including those used to control for confounding(10/48-11/24)
		(*b*) Describe any methods used to examine subgroups and interactions(n/a)
		(*c*) Explain how missing data were addressed(n/a)
		(*d*) *Cohort study*—If applicable, explain how loss to follow-up was addressed<br>*Case-control study*—If applicable, explain how matching of cases and controls was addressed(n/a)<br>*Cross-sectional study*—If applicable, describe analytical methods taking account of sampling strategy(n/a)
		(*e*) Describe any sensitivity analyses(n/a)

Continued on next page

For peer review only - http://bmjopen.bmj.com/site/about/guidelines.xhtml

**Results**		
Participants	13*	(a) Report numbers of individuals at each stage of study—eg numbers potentially eligible, examined for eligibility, confirmed eligible, included in the study, completing follow-up, and analysed(n/a)
		(b) Give reasons for non-participation at each stage(n/a)
		(c) Consider use of a flow diagram(n/a)
Descriptive data	14*	(a) Give characteristics of study participants (eg demographic, clinical, social) and information on exposures and potential confounders(n/a)
		(b) Indicate number of participants with missing data for each variable of interest(n/a)
		(c) *Cohort study*—Summarise follow-up time (eg, average and total amount)(n/a)
Outcome data	15*	*Cohort study*—Report numbers of outcome events or summary measures over time(n/a)
		Case-control study—Report numbers in each exposure category, or summary measures of exposure(n/a)
		Cross-sectional study—Report numbers of outcome events or summary measures(n/a)
Main results	16	(*a*) Give unadjusted estimates and, if applicable, confounder-adjusted estimates and their precision (eg, 95% confidence interval). Make clear which confounders were adjusted for and why they were included(11/38-12/23, 15/26-42)
		(*b*) Report category boundaries when continuous variables were categorized(n/a)
		(*c*) If relevant, consider translating estimates of relative risk into absolute risk for a meaningful time period(16/33-17/18)
Other analyses	17	Report other analyses done—eg analyses of subgroups and interactions, and sensitivity analyses(n/a)
**Discussion**		
Key results	18	Summarise key results with reference to study objectives(17/22-43)
Limitations	19	Discuss limitations of the study, taking into account sources of potential bias or imprecision. Discuss both direction and magnitude of any potential bias(19/22-31)
Interpretation	20	Give a cautious overall interpretation of results considering objectives, limitations, multiplicity of analyses, results from similar studies, and other relevant evidence(17/45-19/20)
Generalisability	21	Discuss the generalisability (external validity) of the study results(n/a)
**Other information**		
Funding	22	Give the source of funding and the role of the funders for the present study and, if applicable, for the original study on which the present article is based(20/9-21)

*Give information separately for cases and controls in case-control studies and, if applicable, for exposed and unexposed groups in cohort and cross-sectional studies.

Note: An Explanation and Elaboration article discusses each checklist item and gives methodological background and published examples of transparent reporting. The STROBE checklist is best used in conjunction with this article (freely available on the Web sites of PLoS Medicine at http://www.plosmedicine.org/, Annals of Internal Medicine at http://www.annals.org/, and Epidemiology at http://www.epidem.com/). Information on the STROBE Initiative is available at www.strobe-statement.org.

For peer review only

For peer review only - http://bmjopen.bmj.com/site/about/guidelines.xhtml

BMJ Open

Effects of global and domestic tobacco control policies on cigarette consumption per capita: An evaluation using monthly data in China

Journal:	*BMJ Open*
Manuscript ID	bmjopen-2018-025092.R2
Article Type:	Research
Date Submitted by the Author:	30-Mar-2019

Complete List of Authors:	Xu, Xiaoxin; Beijing Normal University, School of Social Development and Public Policy<br>Zhang, Xiulan; Beijing Normal University, School of Social Development and Public Policy<br>Hu, Teh-wei; Public Health Institute, Center for International Tobacco Control<br>Miller, Leonard; University of California, School of Social Welfare<br>XU, Mengnan; Beijing Normal University, School of Social Development and Public Policy

**Primary Subject Heading**:	Smoking and tobacco
Secondary Subject Heading:	Global health, Health economics, Health policy
Keywords:	global health, smoking, global and domestic health policies, China, tobacco control

SCHOLARONE™
Manuscripts

Effects of global and domestic tobacco control policies on cigarette consumption per capita: An evaluation using monthly data in China

Word Count: 3882

Xu Xiaoxin, Xiulan Zhang, Teh-wei Hu, Leonard Miller, Mengnan Xu

Authors

Xiaoxin Xu, Ph.D.,

School of Social Development and Public Policy,

Beijing Normal University, China

Email: xuxiaoxin@bnu.edu.cn

Xiulan Zhang, Ph.D.,

School of Social Development and Public Policy,

Beijing Normal University, China

Email: zhang99@bnu.edu.cn

Teh-wei Hu, Ph.D.,

Center for International Tobacco Control,

Public Health Institute, Oakland, CA, USA

Email: thu@berkeley.edu

Leonard S. Miller, Ph.D.,

School of Social Welfare,

For peer review only

University of California, Berkeley, CA, USA

Email: lsmiller2331@gmail.com

Mengnan Xu, B.S.,

School of Social Development and Public Policy,

Beijing Normal University, China

Email: pumpkinggg@126.com

Correspondence: Xiulan Zhang,

School of Social Development and Public Policy, Beijing Normal University, Beijing, China, 19 Xinjiekouwai Street, Haidian District, Beijing, 100875, China. Tel: (86-10) 5880-1512; Fax: (86-10) 5880-0366; Email: zhang99@bnu.edu.cn

For peer review only

For peer review only - http://bmjopen.bmj.com/site/about/guidelines.xhtml

Abstract

Introduction China consumes 44% of the world's cigarettes. Robust tobacco control measures are needed to contain the trend of increasing cigarette consumption. This paper examines the effectiveness of policy interventions introduced in China on reducing the country's tobacco use.

Methods The paper uses data on China's monthly cigarette consumption per capita from January 2000 to June 2017 to estimate the impact of specific policies on China's tobacco consumption. Tobacco consumption is calculated from monthly sales data from the China National Tobacco Corporation and demographic data from the China National Bureau of Statistics. The policies studied include the World Health Organization (WHO) Framework Convention on Tobacco Control (FCTC), national tobacco-related policy changes, and two tobacco tax increases implemented in China during the study period. Segmented regression analysis is used to estimate the immediate effects of the policies studied and changes in the time trends resulted from these policy changes.

Findings The impact of national policy changes in China is almost 20 times greater than the impact of the WHO FCTC treaty itself, and national policy changes in tobacco control are a determining factor in reversing the trend of increasing tobacco consumption in China. The 2015 tax increase, which raised retail cigarette prices, produced both immediate and trend effects, with a total incremental effect 7.8 times that of the 2009 tax increase, which did not result in higher cigarette prices for the consumer.

Interpretations Translating global tobacco control policies into domestic policies will generate a much greater impact on reducing average cigarette consumption, and tobacco

For peer review only - http://bmjopen.bmj.com/site/about/guidelines.xhtml

taxes that are reflected in the retail prices of cigarettes will be more effective in reducing cigarette consumption.

Strengths and limitations of this study

1. To the best of our knowledge, this study is the first systematic evaluation of the impact of both domestic and global tobacco control policies on tobacco consumption in China.
2. The study compares the effectiveness of the global FCTC and domestic policies in reducing cigarette consumption in China over a period of 17.5 years.
3. The data used for the policy evaluation cover the periods from no tobacco control policies in China to the implementation of FCTC policies to the changed national policies to the specific tax increases enacted in China in 2009 and 2015.
4. Using the interrupted time series model, the study examines not only the immediate impact of each policy on tobacco consumption, but also its impact on tobacco consumption trend.
5. The limitations of this study are that the social norm change has not been incorporated into the models, and the cigarette consumption is based on wholesales rather than retails.

For peer review only

Introduction

The 315 million smokers in China consume 44% of the world's cigarettes, and their average consumption is 2.3 times the world average.[1] Tobacco use increases medical expenses by billions.[1] Each year, one million people in China, many of them young, die of tobacco-related diseases.[2] China's rapid economic development in the past 40 years has been accompanied by significant growth in the country's total cigarette consumption. In 2000, the China National Tobacco Corporation (CNTC), the state-owned tobacco monopoly, sold 76.92 billion packs of cigarettes;[3] by 2014, the number had grown to 127.48 billion packs,[4] an increase of 65.8 %.

China signed the World Health Organization (WHO) Framework Convention on Tobacco Control (FCTC) in 2003; the China National People's Congress (CNPC) ratified the treaty in 2005, and China began implementing the FCTC in 2006, indicating that China's government would fulfill its legal obligation in accordance with the treaty.[5] The ratification and implementation of the FCTC provided a moral and legal high ground for advocates on tobacco control[6], although the implementation of specific articles still had a long way to go.[7] In this paper, we refer to the WHO FCTC as an international policy. While the Chinese government has made some effort to control tobacco use, strong interference from the China State Tobacco Monopoly Administration (STMA), which owns CNTC and favors economic concerns over social concerns, has led to slow development and implementation of the full tobacco control policy measures. [8-13]

Between 2006 and 2015, China increased tobacco taxes twice. The first tax increase was introduced in May 2009 and was not reflected directly in cigarette retail prices. As a result, the increase had minimal immediate impact on consumers; it might have more

For peer review only - http://bmjopen.bmj.com/site/about/guidelines.xhtml

long term impact by changing cigarette product structure and consequent raising of average price.[14] The 2009 adjustment raised the ad valorem tax from 45% to 56% at the producer price level for class A cigarettes and from 30% to 36% for class B cigarettes. The new policy also introduced a new 5% ad valorem tax at the wholesale price level.[15 16] The intent of this 2009 adjustment was to raise government revenue from CNTC, China's tobacco producer, not to serve as a tobacco control policy instrument. Under the new scheme, the government forbade changes in the retail prices of cigarettes.[16-18] The policy was introduced primarily to counteract the impact of the global financial crisis on government revenue. Between January and April 2009, China's public revenue decreased 9.9% while public spending increased 31.7%.[19] The financial pressure prompted the government to raise the tobacco tax. In other words, this policy was driven by an economic goal, and because the policy forbade the tobacco industry from adding the tax increase to the retail price of cigarettes, the social goal was not considered at all.[16 17] This outcome was possible because of the unique cigarette pricing mechanism under China's tobacco monopoly system. Both the cigarette allocation price, the price at which the tobacco producers offer cigarettes to the wholesalers, and the wholesale price, the price at which the wholesalers offer cigarettes to retailers, are controlled by STMA. In 2009's tobacco tax adjustment, STMA reduced the wholesale profit margin but maintained the retail price unchanged. In this sense, the 2009 tobacco tax adjustment could be regarded as a profit tax adjustment rather than an excise tax adjustment. The second tobacco tax increase occurred in May 2015. Unlike the 2009 tax adjustment, the 2015 adjustment moved the increase from the tax base at the wholesale price level to the retail price level, a significant step away from the 2009 increase and toward China's

For peer review only - http://bmjopen.bmj.com/site/about/guidelines.xhtml

tobacco control agenda.[20] The 2015 tax increase initiated a 0.10 RMB （0.0146 USD） tax per pack at the wholesale price level and increased the ad valorem tax from 5% to 11%, a 6% point increase also at the wholesale price level. However, this time, the Chinese government allowed the tobacco industry to shift this new tax increase to the retail price, resulting in an estimated 10% increase in the retail price of cigarettes.[21] China's new administration came into power in 2013. With support from its top leader, China's policy direction of tobacco control began to change.[8 22 23]The national policy change began with the anti-corruption campaign, which was aimed at the problem of corruption within the party, state, and business sectors.[24] In November 2013, the government forbade the purchase of cigarettes using public revenue. A month later, additional policies were announced that prohibited state employees/officials from smoking in public places. This significant policy initiative can be considered as a major government effort to change the social norm of smoking habits in China. Figure 1 shows the timeline of major tobacco control policies in China.

Insert Figure 1 Here

One factor that has influenced the trend of increased cigarette consumption in China is the rapid growth of personal income. China has experienced the largest economic transformation in human history. Based on data from the China National Bureau of Statistics (http://data.stats.gov.cn/ks.htm?cn=C01&zb=A0501), following the 1978 economic reform, the Chinese economy grew around 9.5% each year, becoming the second largest economy in the world according to a World Bank report.[25] In recent years,

For peer review only - http://bmjopen.bmj.com/site/about/guidelines.xhtml

China's economic performance has remained at a relatively high level of 7% growth. While the income of people in China also has increased significantly, an increase in cigarette prices has not accompanied the GDP growth, thus making cigarettes more affordable over time.[26 27]

Waiting for China to take robust measures to control its tobacco use, change the social norm and policy landscape, reduce the institutional barriers created by STMA, and counteract the increased consumption of cigarettes resulting from income growth is a long and frustrating process.

The purpose of this paper is to estimate the relative impacts of four tobacco control policy interventions on tobacco consumption in China: first ever international public health treaty, WHO FCTC, the government's 2013 national policy forbidding using general revenue to purchase cigarettes and smoking in public by state employees/officials, and the tax increases of 2009 and 2015, respectively. This is the first study to estimate the combined impact of international and domestic tobacco control policy changes on long-term trends in tobacco consumption in China.

Methods

Data

We used the monthly data on cigarette sales from January 2000 to June 2017, a total of 210 months of data reported by CNTC. Sales data, collected by CNTC, are based on the purchases of retailers, so the exact monthly sales are determined by the dates when retailers buy from cigarette distributors. We extracted these data from *China Tobacco Magazine* and its website (http://www.echinatobacco.com), hosted by CNTC.

For peer review only - http://bmjopen.bmj.com/site/about/guidelines.xhtml

The population data were based on the China Statistical Yearbook, extracted from the website of the China National Bureau of Statistics (http://www.stats.gov.cn/tjsj/ndsj/#). Between 2000 and 2016, China's total population increased by 9.1%. To adjust for the effect of population growth on cigarette sales, this study uses the average packs of cigarettes consumed each month per capita.[28] To estimate policy impacts, we include the GDP growth rate, the timing of policy interventions, and trends initiated by each of the four policy interventions studied here.

During the study period, several tobacco control policies were implemented in China. As discussed in the introduction section, the first was the ratification of WHO FCTC, the implementation of which began in January 2006. In May 2009, China raised cigarette taxes, but the increase was not reflected in the retail price. In 2013, tobacco control received top leadership support. In November, a national policy was issued forbidding government funds from being used to purchase cigarettes for officials, and a month later, in December, the Central Committee of the Communist Party of China (CCCPC) and the State Council jointly issued a policy prohibiting state employees/officials from smoking in public places. This national policy targeted state officials and government agencies. In May 2015, China again raised cigarette taxes and allowed retail prices to rise.

Since the implementation of WHO FCTC, smoke-free policies have been established in different cities or regions of China. The Beijing Municipal Government passed the strictest smoke-free regulation in May 2015. But a national smoke-free law has not yet been passed. Therefore, the effect of smoke-free policies is an unmeasured effect in the model.

For peer review only - http://bmjopen.bmj.com/site/about/guidelines.xhtml

We divided the analysis into five time periods: (1) before the FCTC was implemented (January 2000 to December 2005); (2) between implementation of the FCTC and the first tax policy adjustment (January 2006 to April 2009); (3) between the first tax policy adjustment and implementation of the national policy change (May 2009 to October 2013); (4) between implementation of the national policy change and the second tax policy adjustment (November 2013 to April 2015); and (5) the period after all policies were implemented (May 2015 to June 2017).

Table 1 presents descriptive statistics of the GDP growth rates and average packs of cigarettes consumed during each of the five periods studied.

Table 1 Average cigarette consumption and GDP growth in different periods

Period	Policies	Number of Months	Average Consumption (Pack/month/per capita)	Average GDP Growth (%)
Jan 2000 to Dec 2005	No Policies	72	5.66 (0.57)	9.54 (1.06)
Jan 2006 to Apr 2009	FCTC Only	40	6.78 (1.22)	11.86 (2.02)
May 2009 to Oct 2013	FCTC/Tax1	54	7.43 (1.83)	8.87 (1.11)
Nov 2013 to Apr 2015	FCTC/Tax1/National	18	7.69 (2.14)	7.32 (0.25)
May 2015 to Jun 2017	FCTC/Tax1/National/Tax2	26	7.20 (1.56)	6.81 (0.10)

The GDP growth rates during the 17 years for which we have data reached 14.16% in 2007 and then dropped to 6.7% in 2016. The average growth rate over the analysis period was 9.36%. The decline in GDP growth rates began in 2012.

Patient and Public Involvement

There is no patient and public involvement in data collection.

Statistical Analysis

Segmented regression analysis of interrupted time series is an effective statistical method to evaluate longitudinal effects of time-delimited interventions,[29] and it is widely used in

For peer review only - http://bmjopen.bmj.com/site/about/guidelines.xhtml

assessing policy impact especially where randomization is not feasible.[30] In this model, two parameters are estimated for each intervention studied: level and trend. The level parameter defines the y-intercept, which is the immediate effect of the intervention on the outcome. The time trend interaction with the intervention variable is the rate of change (the slope), which measures the gradual change of the outcomes due to the intervention.[30 31]

We estimated the segmented regression model using SAS AUTOREG procedures to assess the longitudinal impact of tobacco control policies on the average cigarette consumption per month per capita. We estimated levels and trends of the four interventions: FCTC (2005), first taxation (2009), national policies (2013), and second taxation (2015). The monthly pattern of sales was adjusted by the AR parameters in AUTOREG procedure.

Results

Table 2 presents an estimation of the model describing average sales of packs of cigarettes consumed per person per month. Overall, the model is very significant with a total R-squared of 0.9416. The transformed R-squared is 0.995, indicating an extremely high fit of the model and the existence of autocorrelation.

Table 2 Auto-regression model estimate of the per capita monthly cigarette consumption

Maximum-Likelihood Estimates of the Model			
SSE	30.94	DFE	187
MSE	0.165	Root MSE	0.407
SBC	338.86	AIC	261.88
MAE	0.27	AICC	267.81
MAPE	4.04	HQC	293.00
Log Likelihood	-107.94	Observations	210
Total R^2			0.9416
Transformed R^2			0.9950
**Parameter Estimates**			

For peer review only - http://bmjopen.bmj.com/site/about/guidelines.xhtml

Variable	Estimate	t Value	Pr > |t|
Intercept	4.8353	70.45	<.0001
GDP Growth	0.0180	2.10	0.0371
Time	0.0184	32.99	<.0001
FCTC	-0.1101	-2.53	0.0121
FCTC_time	0.0046	2.37	0.0189
Tax1	-0.0462	-1.38	0.1686
Tax1_time	-0.0097	-7.55	<.0001
National	-0.3056	-5.63	<.0001
National_time	0.0078	1.72	0.0871
Tax2	-0.4309	-6.39	<.0001
Tax2_time	-0.0382	-8.30	<.0001
AR1	0.6277	8.87	<.0001
AR2	0.5907	8.04	<.0001
AR3	0.5834	7.83	<.0001
AR4	0.5588	7.33	<.0001
AR5	0.5435	7.00	<.0001
AR6	0.5458	6.85	<.0001
AR7	0.5412	6.72	<.0001
AR8	0.5397	6.72	<.0001
AR9	0.5546	6.98	<.0001
AR10	0.5662	7.23	<.0001
AR11	0.5644	7.32	<.0001
AR12	-0.3572	-4.83	<.0001

The time effect is positive and significant, indicating the urgency to interrupt the trend of increasing cigarette consumption in China to reduce the burden of diseases and death due to smoking.

The parameter estimate shows that GDP growth has a positive and statistically significant impact on monthly cigarette consumption. GDP growth indicates a macro income effect; this effect conforms with the literature that the income elasticity of cigarette consumption is positive.

In terms of the impact of tobacco control policies, implementation of the FCTC in 2006 resulted in an immediate reduction in the number of cigarettes consumed. However, after the initial reduction, consumption again rose over time, indicating that either the tobacco industry developed new strategies to counteract the FCTC policy or consumers resumed the intensity of their smoking habits after the initial reaction to the macro-policy change.

For peer review only - http://bmjopen.bmj.com/site/about/guidelines.xhtml

For peer review only

Similar to what happened after implementation of the international treaty, when the CCCPC and the State Council jointly issued a national policy on state employees/officials and governments in 2013, consumption of cigarettes dropped immediately. The drop following announcement of the national policy was about three times the drop in consumption after implementation of the FCTC. Again, similar to what happened after implementation of the FCTC, the trend after the change in national policy was positive subsequent to the drop, but not statistically significant. This finding shows that while the 2013 national policy changes aimed at changing cigarette-related social norms could immediately impact average sales, the after effect was counteracted by either consumer habits or more aggressive marketing strategies by the tobacco industry.

As for China's two tobacco tax initiatives, the coefficient of the Tax 1 (2009) variable is not statistically significant, as one would have expected. Over time, the tobacco industry restructured its market share but the magnitude of the coefficient of the time and tax interaction term is still very small, though statistically significant. However, the coefficient of the Tax2 (2015) variable and its time trend interaction term are both statistically significant with a magnitude four times larger than the FCTC effect and 40 percent higher than the national policy effect. As shown from the coefficient and its interaction term, the 2015 tobacco tax increase (Tax2) essentially reduced per capita monthly consumption by 0.43 pack initially and then continued to reduce consumption by 0.04 pack per capita per month over time.

The implementation of both tax increases (2009 and 2015) resulted in similar initial effects and time trends. The initial effect of the second (2015) tax increase, aimed at wholesale and retail prices, was about ten times the initial effect of the 2009 tax increase.

For peer review only - http://bmjopen.bmj.com/site/about/guidelines.xhtml

The trend effect of the 2015 policy was about four times the trend effect of the first tax increase. In addition, unmeasured smoke-free model effects might have contributed to the big impact of the 2015 tax increase.

This finding indicates that unless specific policies are targeted at smokers, generalized policies advocating tobacco control may result in some immediate effects, but they won't be able to change the consumption of tobacco over time.

Tax increases are much more effective at changing tobacco consumption than generalized policies. This was true of even the first tax increase (in 2009), which was not reflected in the retail price of cigarettes. When the tax increase was factored into the retail price (in 2015), the impact on cigarette consumption was much larger and was sustained over time.

Figure 2 presents China's average monthly packs of cigarettes consumed per capita per year from 2000-2017 with and without accounting for tobacco control policies. Before the FCTC policy was implemented, between 2000 and 2005, average consumption increased from 5.1 packs to 6.3 packs per capita per month, an increase of 23.5% in six years.

Between 2006 and 2013, monthly cigarette consumption grew from 6.4 packs to 7.7 packs, an increase of 20.3% in seven years. Consumption then began to decrease in 2013. By the end of 2016, the average number of packs of cigarettes consumed monthly had dropped from 7.7 to 7.2, a 6.5% decrease in three years. Without the tax increase, average consumption was predicted at 8.6 packs, 16.3% higher than with the tax increase.

The 2013 policy announcements by the Chinese national government changed smoking-related social norms. Combined with the global FCTC intervention and the first tax increase (2009), the growing trend of cigarette consumption per month per capita in

For peer review only - http://bmjopen.bmj.com/site/about/guidelines.xhtml

China finally began to decline, and the second tax increase (2015) had a much bigger impact on the downward trend.

Insert Figure 2 Here.

Based on the model estimates, we calculated the total impact of various policies on the average number of packs of cigarettes consumed per month. Table 3 presents the percentage change in average monthly consumption of cigarettes, with and without tobacco control policies. The percentage effect is calculated as follows:

$$\% \text{ Change} = (Y\hat{}_{policies=1} - Y\hat{}_{policies=0})/Y\hat{}_{policies=0}$$

Table 3 Impact of tobacco control policies on average monthly cigarette consumption per capita

Policy Period	Predicted Monthly Consumption in Packs of Cigarettes with Policies ($Y\hat{}_{policies=1}$)	Predicted Monthly Consumption in Packs of Cigarettes without Policies ($Y\hat{}_{policies=0}$)	% Change ($Y\hat{}_{policies=1}$ - $Y\hat{}_{policies=0}$) /$Y\hat{}_{policies=0}$	Incremental Change
No Policies	5.680	5.680	0.00%	0.00%
FCTC Only	6.737	6.754	-0.25%	-0.25%
FCTC/Tax1	7.450	7.566	-1.54%	-1.29%
FCTC/Tax1/National	7.668	8.202	-6.51%	-4.97%
FCTC/Tax1/National/Tax2	7.176	8.598	-16.54%	-10.03%

Table 3 shows that during the 40-month period when only the FCTC policy was in effect (January 2006 through April 2009), average cigarette consumption dropped 0.25%, due mainly to the initial impact of the FCTC.

During the 54-month period that includes implementation of the FCTC and the first tax increase (May 2009 through October 2013), average consumption dropped 1.54%, and

For peer review only - http://bmjopen.bmj.com/site/about/guidelines.xhtml

the incremental effect of the 2009 tax increase was -1.29%, indicating a very limited effect when the tax increase was not factored into the retail price.

During the 18-month period following issuance of the national policies (November 2013 through April 2015), but prior to the second tax increase instituted in May 2015, the average consumption of cigarettes dropped 6.51%, and the national policy changes alone reduced monthly consumption by 4.97%.

After the second tax increase announced in May 2015, a big decline occurred in cigarette consumption. In the 26-month period following this tax increase (May 2015 through June 2017), the average consumption of cigarettes dropped 16.54%, due mainly to the effect of the second tax increase, which alone brought down average monthly consumption by 10.03%.

Table 4 presents the predicted effects of the four policies studied on cigarette consumption measured in million packs.

Table 4 Impact of tobacco control policies on cigarette consumption in million packs

Period	FCTC	1st Taxation	National	2nd Taxation	All Policies
Jan 2006 to Apr 2009	893	0	0	0	893
May 2009 to Oct 2013	1,377	7,106	0	0	8,484
Nov 2013 to Apr 2015	505	2,606	10,042	0	13,153
May 2015 to Jun 2017	771	3,980	15,336	30,949	51,036
Total	3,547	13,693	25,377	30,949	73,567

Between January 2006 and June 2017, China consumed 1.348 trillion packs of cigarettes. The reduction in total consumption attributable to the policy changes was 73.6 billion packs, which is 5.18% of the sales predicted without policy interventions.

Implementation of the FCTC decreased consumption by 3.5 billion packs, the first tax

For peer review only - http://bmjopen.bmj.com/site/about/guidelines.xhtml

increase (2009) reduced consumption by 13.7 billion, the national policy announcements decreased it by 25.4 billion packs, and the largest reduction came from the second tax increase (2015), including unmeasured local smoke-free policies--almost 31 billion packs in just 26 months.

From announcement of the 2013 national policy change through June 2017, China reduced the sales of cigarettes by 64.2 billion packs, a 12.57% reduction in the average consumption of cigarettes in China.

Discussion

The WHO FCTC, an international treaty, aims to provide a roadmap to address the global tobacco epidemic using effective measures and strategies. China ratified the treaty in November 2005 and began implementation in January 2006.

Calculated from Table 3, this study finds that the impact of national policy changes has been almost 20 times larger than the impact of the WHO FCTC treaty itself, and that national tobacco control policy changes in China have been a determining factor in reversing the increasing trend of tobacco consumption. In other words, implementing an international treaty requires national policy change to achieve the goal of reducing tobacco consumption. Ratification of the treaty alone without domestic policy implementation will have a very minimal effect.

The process of integrating global policy with domestic policy change took exactly 10 years in China (November 2003 to November 2013).[32] Our study finds that after the immediate effects of the policy changes were noted, the powerful STMA developed countermeasures to dilute the impact of the policy changes. This finding confirms the

For peer review only - http://bmjopen.bmj.com/site/about/guidelines.xhtml

challenges faced by and the persistence required for the global and national tobacco control communities. [7 33]

Between 2006 and 2013, although the government raised the tobacco tax in May 2009, the economic goal of increasing government revenue overpowered the social goal of reducing tobacco consumption. The tax increase did not result in higher cigarette prices for the consumer, thus minimizing the impact of this policy on consumption.[17 34]

When the 2015 tax policy raised retail cigarette prices, both immediate and trend effects were very significant, and the total incremental effect was 7.8 times that of the 2009 tax increase (the ratio is calculated as the incremental effect of tax2 which is the difference of FCTC/Tax1/National/Tax2 and FCTC/Tax1/National presented in Table 3: -16.54% and -6.51%=-10.03%, and the incremental effect of tax1 is the difference of FCTC/Tax1 and FCTC Only: -1.54% and -0.25%=-1.29%. The ratio of incremental effect tax2 and tax1 is -10.03% and -1.29%=7.8). This finding indicates that tobacco control policies should be more robust and target consumers more directly through higher prices and tougher smoke-free regulations. Because China has no national smoke-free law, and the impact of various local smoke-free regulations on national cigarette consumption is difficult to measure, the impact of taxation policy includes unmeasured effects of local smoke-free policies.

This study finds a significant positive income effect on consumption, which indicates that cigarettes have become more affordable. A recent study shows that between 2001 and 2016, the affordability of cigarettes in China increased 1.85 times. It is important to continue to raise the tobacco tax to offset the affordability influence on cigarette consumption.[27]

For peer review only - http://bmjopen.bmj.com/site/about/guidelines.xhtml

This study shows empirically that raising the tobacco tax through increasing retail prices is the most effective tobacco control policy instrument among the few policies implemented in China. Currently China has a relatively low cigarette tax rate, 56% of the retail price.[20] The WHO guideline for an effective tobacco control benchmark is a tax rate of 75% of the retail price.[35] Comparing China's tax rate with the WHO guideline reveals that China has a lot of room to raise its tax on tobacco. Raising the price of cigarettes will save lives, reduce smoking-related medical costs, and generate additional government revenue.

This study has some limitations. During the study period, the social norm of smoking in China has changed significantly because of the tobacco control policy changes and increased awareness of the negative health effects of smoking. This change in the social norm has not been incorporated into the model estimates. In addition, the cigarette consumption data are based on wholesale data rather than retail data. However, because the study is based on time series monthly data, and the retailers are normally not holding a large inventory, the impact of this data source on the findings is limited.

Contributorship statement

Xiaoxin Xu: Conducted literature review, participated in data collection and manuscript writing; Xiulan Zhang: Directed and verified data collection, estimated the models and drafted the findings of the models and the discussions; Teh-wei Hu: Reviewed the models and findings, participated in writing and discussions; Leonard S. Miller: Reviewed the models and findings, participated in drafting the main findings of the models; and

For peer review only - http://bmjopen.bmj.com/site/about/guidelines.xhtml

Mengnan Xu: Participated in data collection and verification, participated in literature review.

Competing Interest

The authors declare that they have no competing interests.

Funding

Bill & Melinda Gates Foundation

Role of the funding source

The funder had no role in the study design, collection, analysis, or interpretation of the data, writing the manuscript, or the decision to submit the paper for publication. The corresponding author had full access to all data in the study and had final responsibility for the decision to submit for publication.

Data sharing statement

Extra data is available by emailing Xiulan Zhang.

Acknowledgement

The authors would like to thank Ms. D. Lynne Kaltreider for her proofreading of the manuscript.

References

1. The Bill China Cannot Afford: Health, Economic and Social Costs of China's Tobacco Epidemic. Manila: World Health Organization Regional Office for the Western Pacific, 2017.

For peer review only - http://bmjopen.bmj.com/site/about/guidelines.xhtml

2. Chen Z, Peto R, Zhou M, et al. Contrasting male and female trends in tobacco-attributed mortality in China: evidence from successive nationwide prospective cohort studies. *The Lancet* 2015;386(10002):1447-56. doi: https://doi.org/10.1016/S0140-6736(15)00340-2

3. Development of China's tobacco industry in 2000 (in Chinese). *China Tobacco* 2001(4)

4. Analysis of China's tobacco market in 2016 (in Chinese). *China Tobacco* 2017(5):62-65.

5. Yang G. Introduction: China and the Negotiation of WHO FCTC. In: Yang G, ed. Tobacco control in China. Singapore: Springer 2018:1-16

6. Huang J. Public interest litigation and tobacco control in China. In: Yang G, ed. Tobacco control in China. Singapore: Springer 2018:119-40

7. Xiao D, Bai C-X, Chen Z-M, et al. Implementation of the World Health Organization Framework Convention on Tobacco Control in China: An arduous and long-term task. *Cancer* 2015;121(S17):3061-68. doi: https://doi.org/10.1002/cncr.29608

8. Yang G, Wang Y, Wu Y, et al. The road to effective tobacco control in China. *The Lancet* 2015;385(9972):1019-28. doi: https://doi.org/10.1016/S0140-6736(15)60174-X

9. Chen MH. Economic concerns hamper tobacco control in China. *The Lancet* 2007;370(9589):729-30. doi: Http://doi.org/10.1016/S0140-6736(07)61359-2

10. Clarke H, Tan BJ. Tobacco Use Control Policies in China. *Economic Papers: A Journal of Applied Economics and Policy* 2011;30(4):490-96. doi: https://doi.org/10.1111/j.1759-3441.2011.00132.x

11. Pratt CB. China's tobacco industry's communication practices: Paradoxes and proposals for public policymaking. *Public Relations Review* 2011;37(3):318-20. doi: https://doi.org/10.1016/j.pubrev.2011.03.010

12. Huang Y. China's position in negotiating the Framework Convention on Tobacco Control and the revised International Health Regulations. *Public Health* 2014;128(2):161-66. doi: https://doi.org/10.1016/j.puhe.2013.08.003

For peer review only

13. Jin J. Why FCTC Policies Have Not Been Implemented in China: Domestic Dynamics and Tobacco Governance. *Journal of Health Politics, Policy and Law* 2014;39(3):633-66. doi: https://doi.org/10.1215/03616878-2682630

14. Zheng R, Gao S, Hu T-W. Tobacco Tax and Tobacco Control: Global Experience and Its application in China (in Chinese). *Finance & Trade Economics* 2013;34(3):44-53.

15. Ministry of Finance, State Administration of Taxation. Notice on Adjustment to Excise Tax on Tobacco Products(Taxation Document No.2009-84)(in Chinese), 2009.

16. Hu T-w, Mao Z, Shi J. Recent tobacco tax rate adjustment and its potential impact on tobacco control in China. *Tobacco Control* 2010;19(1):80-82. doi: http://dx.doi.org/10.1136/tc.2009.032631

17. Shi J, Hu T-w, Mao Z. Economic Effect Analysis of China's Tobacco Excise Tax Reform(in Chinese). *Review on Finance and Economics* 2011(1):52-59.

18. Gao S, Zheng R, Hu T-w. Can increases in the cigarette tax rate be linked to cigarette retail prices? Solving mysteries related to the cigarette pricing mechanism in China. *Tobacco Control* 2012;21(6):560-62. doi: http://dx.doi.org/10.1136/tobaccocontrol-2011-050027

19. Ministry of Finance. Overview of China's fiscal revenue and expenditure in April 2009 (in Chinese) Beijing: Ministry of Finace of the People's Republic of China; 2009 [Available from: http://gks.mof.gov.cn/zhengfuxinxi/tongjishuju/200905/t20090514_157614.html accessed March 27 2019.

20. Hu T-w, Zhang X, Zheng R. China has raised the tax on cigarettes: what's next? *Tobacco Control* 2015;25(6):609-11. doi: http://dx.doi.org/10.1136/tobaccocontrol-2015-052534

21. Zheng R, Wang Y, Hu X. Tobacco tax: theory, system design, and policy practice (in Chinese). *Financial Minds* 2016;1(6):5-30.

22. Mackay J. China: the tipping point in tobacco control. *British Medical Bulletin* 2016;120(1):15-25. doi: https://doi.org/10.1093/bmb/ldw043

For peer review only

23. Alcorn T. Winds shift for tobacco control in China. *The Lancet Respiratory Medicine* 2013;1(9):679-80. doi: Http://doi.org/10.1016/S2213-2600(13)70236-4

24. Wedeman A. Xi Jinping's hunt: Anti-corruption campaign or factional purge? *Modern China Studies* 2017;24(2):35-94.

25. World Bank. World Development Report 2012 : Gender Equality and Development. Washington, DC: The World Bank Group, 2012.

26. Wang L, Yan Y, Yi N, et al. Cigarette consumer price and affordability in China: results from 2015 China adult survey (in Chinese). *Chinese Journal of Epidemiology* 2017;38(1):69-72.

27. Zheng R, Wang Y, Hu X, et al. Cigarette affordability in China: 2001-2016. Washington, DC.: World Bank, 2017.

28. Hu T-w, Sung H-Y, Keeler TE. Reducing cigarette consumption in California: tobacco taxes vs an anti-smoking media campaign. *American Journal of Public Health* 1995;85(9):1218-22. doi: Http://doi.org/10.2105/ajph.85.9.1218

29. McDowall D, McCleary R, Meidinger EE, et al. Interrupted Time Series Analysis. Thousand Oaks, CA: Sage Publications, Inc 1980.

30. Wagner AK, Soumerai SB, Zhang F, et al. Segmented regression analysis of interrupted time series studies in medication use research. *Journal of Clinical Pharmacy and Therapeutics* 2002;27(4):299-309. doi: Http://doi.org/10.1046/j.1365-2710.2002.00430.x

31. Zombré D, De Allegri M, Ridde V. Immediate and sustained effects of user fee exemption on healthcare utilization among children under five in Burkina Faso: A controlled interrupted time-series analysis. *Social Science & Medicine* 2017;179(Supplement C):27-35. doi: https://doi.org/10.1016/j.socscimed.2017.02.027

32. Eight years' implemention of World Heath Organization Framework Convention on Tobacco Control(in Chinese) 2013 [Available from: http://www.chinacdc.cn/jkzt/jkcj/sthd_3844/slhd_4153/201305/t20130515_80977.htm accessed 12-31 2017.

For peer review only - http://bmjopen.bmj.com/site/about/guidelines.xhtml

33. Hu T-w, Lee AH, Mao Z. WHO Framework Convention on Tobacco Control in China: Barriers, challenges and recommendations. *Global Health Promotion* 2013;20(4):13-22. doi: Http://doi.org/10.1177/1757975913501910

34. Gao S, Zheng R. Price, tax and cigarette smoking: Simulations of China's tobacco tax policy. *Frontiers of Economics in China* 2012;7(4):604-26.

35. World Health Organization. WHO Technical Manual on Tobacco Tax Administration. Geneva, Switzerland: World Health Organization, 2010.

For peer review only

Figure 1 Timeline of tobacco control policies in China

For peer review only

For peer review only - http://bmjopen.bmj.com/site/about/guidelines.xhtml

Figure 2 Estimated monthly average packs of cigarettes consumed in China per capita with and without accounting for tobacco control policies, 2000-2017. Dotted red line (without policy), trend without tobacco control policies; Blue line(with policy), trend with tobacco control policies. Both lines are predicted values from the time-series model parameters.

For peer review only

For peer review only - http://bmjopen.bmj.com/site/about/guidelines.xhtml

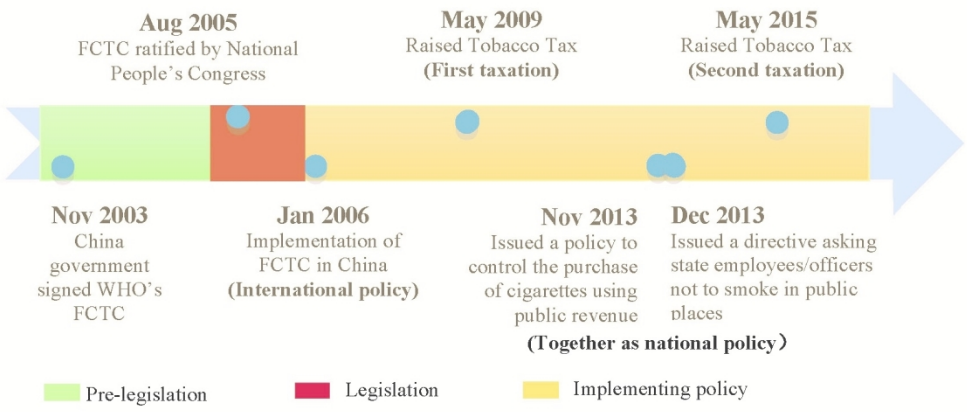

Figure 1 Timeline of tobacco control policies in China

461x196mm (96 x 96 DPI)

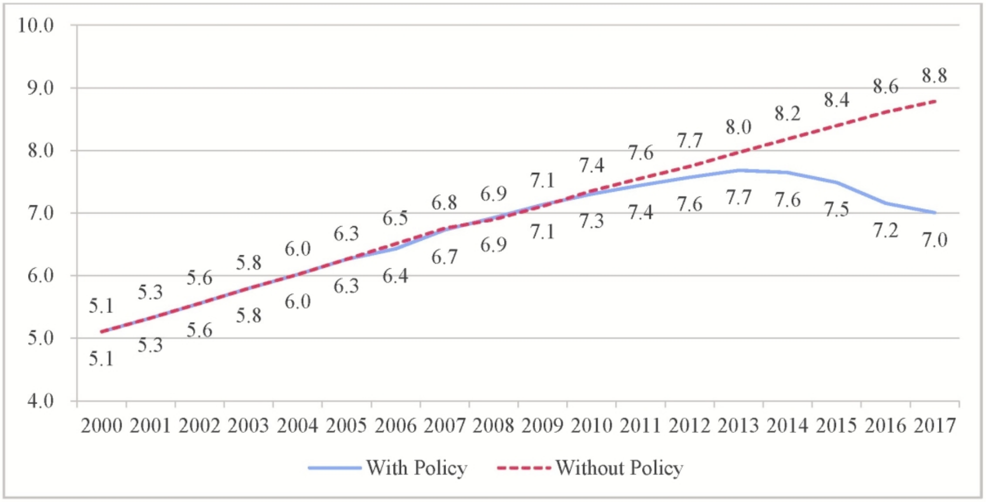

Figure 2 Estimated monthly average packs of cigarettes consumed in China per capita with and without accounting for tobacco control policies, 2000-2017. Dotted red line (without policy), trend without tobacco control policies; Blue line(with policy), trend with tobacco control policies. Both lines are predicted values from the time-series model parameters.

546x284mm (96 x 96 DPI)

STROBE Statement—checklist of items that should be included in reports of observational studies
(Shown in form of a/b, a means page number, and b means line number; n/a means not applicable.)

	Item No	Recommendation
**Title and abstract**	1	(*a*) Indicate the study's design with a commonly used term in the title or the abstract (1/4-7, 3/15-33)
		(*b*) Provide in the abstract an informative and balanced summary of what was done and what was found(3/35-52&4/3-6)
**Introduction**		
Background/rationale	2	Explain the scientific background and rationale for the investigation being reported(6/24-8/22)
Objectives	3	State specific objectives, including any prespecified hypotheses(8/24-38)
**Methods**		
Study design	4	Present key elements of study design early in the paper (n/a)
Setting	5	Describe the setting, locations, and relevant dates, including periods of recruitment, exposure, follow-up, and data collection(n/a)
Participants	6	(*a*) *Cohort study*—Give the eligibility criteria, and the sources and methods of selection of participants. Describe methods of follow-up(n/a)<br>*Case-control study*—Give the eligibility criteria, and the sources and methods of case ascertainment and control selection. Give the rationale for the choice of cases and controls(n/a)<br>*Cross-sectional study*—Give the eligibility criteria, and the sources and methods of selection of participants(n/a)
		(*b*) *Cohort study*—For matched studies, give matching criteria and number of exposed and unexposed(n/a)<br>*Case-control study*—For matched studies, give matching criteria and the number of controls per case(n/a)
Variables	7	Clearly define all outcomes, exposures, predictors, potential confounders, and effect modifiers. Give diagnostic criteria, if applicable(8/45-10/39)
Data sources/ measurement	8*	For each variable of interest, give sources of data and details of methods of assessment (measurement). Describe comparability of assessment methods if there is more than one group(8/45-10/13)
Bias	9	Describe any efforts to address potential sources of bias(n/a)
Study size	10	Explain how the study size was arrived at(n/a)
Quantitative variables	11	Explain how quantitative variables were handled in the analyses. If applicable, describe which groupings were chosen and why(8/45-9/17)
Statistical methods	12	(*a*) Describe all statistical methods, including those used to control for confounding(10/48-11/24)
		(*b*) Describe any methods used to examine subgroups and interactions(n/a)
		(*c*) Explain how missing data were addressed(n/a)
		(*d*) *Cohort study*—If applicable, explain how loss to follow-up was addressed<br>*Case-control study*—If applicable, explain how matching of cases and controls was addressed(n/a)<br>*Cross-sectional study*—If applicable, describe analytical methods taking account of sampling strategy(n/a)
		(*e*) Describe any sensitivity analyses(n/a)

Continued on next page

For peer review only - http://bmjopen.bmj.com/site/about/guidelines.xhtml

For peer review only

**Results**		
Participants	13*	(a) Report numbers of individuals at each stage of study—eg numbers potentially eligible, examined for eligibility, confirmed eligible, included in the study, completing follow-up, and analysed(n/a)
		(b) Give reasons for non-participation at each stage(n/a)
		(c) Consider use of a flow diagram(n/a)
Descriptive data	14*	(a) Give characteristics of study participants (eg demographic, clinical, social) and information on exposures and potential confounders(n/a)
		(b) Indicate number of participants with missing data for each variable of interest(n/a)
		(c) *Cohort study*—Summarise follow-up time (eg, average and total amount)(n/a)
Outcome data	15*	*Cohort study*—Report numbers of outcome events or summary measures over time(n/a)
		Case-control study—Report numbers in each exposure category, or summary measures of exposure(n/a)
		Cross-sectional study—Report numbers of outcome events or summary measures(n/a)
Main results	16	(*a*) Give unadjusted estimates and, if applicable, confounder-adjusted estimates and their precision (eg, 95% confidence interval). Make clear which confounders were adjusted for and why they were included(11/38-12/23, 15/26-42)
		(*b*) Report category boundaries when continuous variables were categorized(n/a)
		(*c*) If relevant, consider translating estimates of relative risk into absolute risk for a meaningful time period(16/33-17/18)
Other analyses	17	Report other analyses done—eg analyses of subgroups and interactions, and sensitivity analyses(n/a)
**Discussion**		
Key results	18	Summarise key results with reference to study objectives(17/22-43)
Limitations	19	Discuss limitations of the study, taking into account sources of potential bias or imprecision. Discuss both direction and magnitude of any potential bias(19/22-31)
Interpretation	20	Give a cautious overall interpretation of results considering objectives, limitations, multiplicity of analyses, results from similar studies, and other relevant evidence(17/45-19/20)
Generalisability	21	Discuss the generalisability (external validity) of the study results(n/a)
**Other information**		
Funding	22	Give the source of funding and the role of the funders for the present study and, if applicable, for the original study on which the present article is based(20/9-21)

*Give information separately for cases and controls in case-control studies and, if applicable, for exposed and unexposed groups in cohort and cross-sectional studies.

Note: An Explanation and Elaboration article discusses each checklist item and gives methodological background and published examples of transparent reporting. The STROBE checklist is best used in conjunction with this article (freely available on the Web sites of PLoS Medicine at http://www.plosmedicine.org/, Annals of Internal Medicine at http://www.annals.org/, and Epidemiology at http://www.epidem.com/). Information on the STROBE Initiative is available at www.strobe-statement.org.

For peer review only - http://bmjopen.bmj.com/site/about/guidelines.xhtml
